# Performance of pile-up mitigation techniques for jets in $pp$ collisions at $\sqrt{s}=8$ TeV using the ATLAS detector

**DOI:** 10.1140/epjc/s10052-016-4395-z

**Published:** 2016-10-27

**Authors:** G. Aad, B. Abbott, J. Abdallah, O. Abdinov, R. Aben, M. Abolins, O. S. AbouZeid, H. Abramowicz, H. Abreu, R. Abreu, Y. Abulaiti, B. S. Acharya, L. Adamczyk, D. L. Adams, J. Adelman, S. Adomeit, T. Adye, A. A. Affolder, T. Agatonovic-Jovin, J. Agricola, J. A. Aguilar-Saavedra, S. P. Ahlen, F. Ahmadov, G. Aielli, H. Akerstedt, T. P. A. Åkesson, A. V. Akimov, G. L. Alberghi, J. Albert, S. Albrand, M. J. Alconada Verzini, M. Aleksa, I. N. Aleksandrov, C. Alexa, G. Alexander, T. Alexopoulos, M. Alhroob, G. Alimonti, L. Alio, J. Alison, S. P. Alkire, B. M. M. Allbrooke, P. P. Allport, A. Aloisio, A. Alonso, F. Alonso, C. Alpigiani, A. Altheimer, B. Alvarez Gonzalez, D. Álvarez Piqueras, M. G. Alviggi, B. T. Amadio, K. Amako, Y. Amaral Coutinho, C. Amelung, D. Amidei, S. P. Amor Dos Santos, A. Amorim, S. Amoroso, N. Amram, G. Amundsen, C. Anastopoulos, L. S. Ancu, N. Andari, T. Andeen, C. F. Anders, G. Anders, J. K. Anders, K. J. Anderson, A. Andreazza, V. Andrei, S. Angelidakis, I. Angelozzi, P. Anger, A. Angerami, F. Anghinolfi, A. V. Anisenkov, N. Anjos, A. Annovi, M. Antonelli, A. Antonov, J. Antos, F. Anulli, M. Aoki, L. Aperio Bella, G. Arabidze, Y. Arai, J. P. Araque, A. T. H. Arce, F. A. Arduh, J.-F. Arguin, S. Argyropoulos, M. Arik, A. J. Armbruster, O. Arnaez, V. Arnal, H. Arnold, M. Arratia, O. Arslan, A. Artamonov, G. Artoni, S. Asai, N. Asbah, A. Ashkenazi, B. Åsman, L. Asquith, K. Assamagan, R. Astalos, M. Atkinson, N. B. Atlay, K. Augsten, M. Aurousseau, G. Avolio, B. Axen, M. K. Ayoub, G. Azuelos, M. A. Baak, A. E. Baas, M. J. Baca, C. Bacci, H. Bachacou, K. Bachas, M. Backes, M. Backhaus, P. Bagiacchi, P. Bagnaia, Y. Bai, T. Bain, J. T. Baines, O. K. Baker, E. M. Baldin, P. Balek, T. Balestri, F. Balli, E. Banas, Sw. Banerjee, A. A. E. Bannoura, H. S. Bansil, L. Barak, E. L. Barberio, D. Barberis, M. Barbero, T. Barillari, M. Barisonzi, T. Barklow, N. Barlow, S. L. Barnes, B. M. Barnett, R. M. Barnett, Z. Barnovska, A. Baroncelli, G. Barone, A. J. Barr, F. Barreiro, J. Barreiro Guimarães da Costa, R. Bartoldus, A. E. Barton, P. Bartos, A. Basalaev, A. Bassalat, A. Basye, R. L. Bates, S. J. Batista, J. R. Batley, M. Battaglia, M. Bauce, F. Bauer, H. S. Bawa, J. B. Beacham, M. D. Beattie, T. Beau, P. H. Beauchemin, R. Beccherle, P. Bechtle, H. P. Beck, K. Becker, M. Becker, S. Becker, M. Beckingham, C. Becot, A. J. Beddall, A. Beddall, V. A. Bednyakov, C. P. Bee, L. J. Beemster, T. A. Beermann, M. Begel, J. K. Behr, C. Belanger-Champagne, W. H. Bell, G. Bella, L. Bellagamba, A. Bellerive, M. Bellomo, K. Belotskiy, O. Beltramello, O. Benary, D. Benchekroun, M. Bender, K. Bendtz, N. Benekos, Y. Benhammou, E. Benhar Noccioli, J. A. Benitez Garcia, D. P. Benjamin, J. R. Bensinger, S. Bentvelsen, L. Beresford, M. Beretta, D. Berge, E. Bergeaas Kuutmann, N. Berger, F. Berghaus, J. Beringer, C. Bernard, N. R. Bernard, C. Bernius, F. U. Bernlochner, T. Berry, P. Berta, C. Bertella, G. Bertoli, F. Bertolucci, C. Bertsche, D. Bertsche, M. I. Besana, G. J. Besjes, O. Bessidskaia Bylund, M. Bessner, N. Besson, C. Betancourt, S. Bethke, A. J. Bevan, W. Bhimji, R. M. Bianchi, L. Bianchini, M. Bianco, O. Biebel, D. Biedermann, S. P. Bieniek, M. Biglietti, J. Bilbao De Mendizabal, H. Bilokon, M. Bindi, S. Binet, A. Bingul, C. Bini, S. Biondi, C. W. Black, J. E. Black, K. M. Black, D. Blackburn, R. E. Blair, J. -B. Blanchard, J. E. Blanco, T. Blazek, I. Bloch, C. Blocker, W. Blum, U. Blumenschein, G. J. Bobbink, V. S. Bobrovnikov, S. S. Bocchetta, A. Bocci, C. Bock, M. Boehler, J. A. Bogaerts, D. Bogavac, A. G. Bogdanchikov, C. Bohm, V. Boisvert, T. Bold, V. Boldea, A. S. Boldyrev, M. Bomben, M. Bona, M. Boonekamp, A. Borisov, G. Borissov, S. Borroni, J. Bortfeldt, V. Bortolotto, K. Bos, D. Boscherini, M. Bosman, J. Boudreau, J. Bouffard, E. V. Bouhova-Thacker, D. Boumediene, C. Bourdarios, N. Bousson, A. Boveia, J. Boyd, I. R. Boyko, I. Bozic, J. Bracinik, A. Brandt, G. Brandt, O. Brandt, U. Bratzler, B. Brau, J. E. Brau, H. M. Braun, S. F. Brazzale, W. D. Breaden Madden, K. Brendlinger, A. J. Brennan, L. Brenner, R. Brenner, S. Bressler, K. Bristow, T. M. Bristow, D. Britton, D. Britzger, F. M. Brochu, I. Brock, R. Brock, J. Bronner, G. Brooijmans, T. Brooks, W. K. Brooks, J. Brosamer, E. Brost, J. Brown, P. A. Bruckman de Renstrom, D. Bruncko, R. Bruneliere, A. Bruni, G. Bruni, M. Bruschi, N. Bruscino, L. Bryngemark, T. Buanes, Q. Buat, P. Buchholz, A. G. Buckley, S. I. Buda, I. A. Budagov, F. Buehrer, L. Bugge, M. K. Bugge, O. Bulekov, D. Bullock, H. Burckhart, S. Burdin, B. Burghgrave, S. Burke, I. Burmeister, E. Busato, D. Büscher, V. Büscher, P. Bussey, J. M. Butler, A. I. Butt, C. M. Buttar, J. M. Butterworth, P. Butti, W. Buttinger, A. Buzatu, A. R. Buzykaev, S. Cabrera Urbán, D. Caforio, V. M. Cairo, O. Cakir, N. Calace, P. Calafiura, A. Calandri, G. Calderini, P. Calfayan, L. P. Caloba, D. Calvet, S. Calvet, R. Camacho Toro, S. Camarda, P. Camarri, D. Cameron, R. Caminal Armadans, S. Campana, M. Campanelli, A. Campoverde, V. Canale, A. Canepa, M. Cano Bret, J. Cantero, R. Cantrill, T. Cao, M. D. M. Capeans Garrido, I. Caprini, M. Caprini, M. Capua, R. Caputo, R. Cardarelli, F. Cardillo, T. Carli, G. Carlino, L. Carminati, S. Caron, E. Carquin, G. D. Carrillo-Montoya, J. R. Carter, J. Carvalho, D. Casadei, M. P. Casado, M. Casolino, E. Castaneda-Miranda, A. Castelli, V. Castillo Gimenez, N. F. Castro, P. Catastini, A. Catinaccio, J. R. Catmore, A. Cattai, J. Caudron, V. Cavaliere, D. Cavalli, M. Cavalli-Sforza, V. Cavasinni, F. Ceradini, B. C. Cerio, K. Cerny, A. S. Cerqueira, A. Cerri, L. Cerrito, F. Cerutti, M. Cerv, A. Cervelli, S. A. Cetin, A. Chafaq, D. Chakraborty, I. Chalupkova, P. Chang, J. D. Chapman, D. G. Charlton, C. C. Chau, C. A. Chavez Barajas, S. Cheatham, A. Chegwidden, S. Chekanov, S. V. Chekulaev, G. A. Chelkov, M. A. Chelstowska, C. Chen, H. Chen, K. Chen, L. Chen, S. Chen, X. Chen, Y. Chen, H. C. Cheng, Y. Cheng, A. Cheplakov, E. Cheremushkina, R. Cherkaoui El Moursli, V. Chernyatin, E. Cheu, L. Chevalier, V. Chiarella, G. Chiarelli, J. T. Childers, G. Chiodini, A. S. Chisholm, R. T. Chislett, A. Chitan, M. V. Chizhov, K. Choi, S. Chouridou, B. K. B. Chow, V. Christodoulou, D. Chromek-Burckhart, J. Chudoba, A. J. Chuinard, J. J. Chwastowski, L. Chytka, G. Ciapetti, A. K. Ciftci, D. Cinca, V. Cindro, I. A. Cioara, A. Ciocio, Z. H. Citron, M. Ciubancan, A. Clark, B. L. Clark, P. J. Clark, R. N. Clarke, W. Cleland, C. Clement, Y. Coadou, M. Cobal, A. Coccaro, J. Cochran, L. Coffey, J. G. Cogan, L. Colasurdo, B. Cole, S. Cole, A. P. Colijn, J. Collot, T. Colombo, G. Compostella, P. Conde Muiño, E. Coniavitis, S. H. Connell, I. A. Connelly, S. M. Consonni, V. Consorti, S. Constantinescu, C. Conta, G. Conti, F. Conventi, M. Cooke, B. D. Cooper, A. M. Cooper-Sarkar, T. Cornelissen, M. Corradi, F. Corriveau, A. Corso-Radu, A. Cortes-Gonzalez, G. Cortiana, G. Costa, M. J. Costa, D. Costanzo, D. Côté, G. Cottin, G. Cowan, B. E. Cox, K. Cranmer, G. Cree, S. Crépé-Renaudin, F. Crescioli, W. A. Cribbs, M. Crispin Ortuzar, M. Cristinziani, V. Croft, G. Crosetti, T. Cuhadar Donszelmann, J. Cummings, M. Curatolo, C. Cuthbert, H. Czirr, P. Czodrowski, S. D’Auria, M. D’Onofrio, M. J. Da Cunha Sargedas De Sousa, C. Da Via, W. Dabrowski, A. Dafinca, T. Dai, O. Dale, F. Dallaire, C. Dallapiccola, M. Dam, J. R. Dandoy, N. P. Dang, A. C. Daniells, M. Danninger, M. Dano Hoffmann, V. Dao, G. Darbo, S. Darmora, J. Dassoulas, A. Dattagupta, W. Davey, C. David, T. Davidek, E. Davies, M. Davies, P. Davison, Y. Davygora, E. Dawe, I. Dawson, R. K. Daya-Ishmukhametova, K. De, R. de Asmundis, A. De Benedetti, S. De Castro, S. De Cecco, N. De Groot, P. de Jong, H. De la Torre, F. De Lorenzi, L. De Nooij, D. De Pedis, A. De Salvo, U. De Sanctis, A. De Santo, J. B. De Vivie De Regie, W. J. Dearnaley, R. Debbe, C. Debenedetti, D. V. Dedovich, I. Deigaard, J. Del Peso, T. Del Prete, D. Delgove, F. Deliot, C. M. Delitzsch, M. Deliyergiyev, A. Dell’Acqua, L. Dell’Asta, M. Dell’Orso, M. Della Pietra, D. della Volpe, M. Delmastro, P. A. Delsart, C. Deluca, D. A. DeMarco, S. Demers, M. Demichev, A. Demilly, S. P. Denisov, D. Derendarz, J. E. Derkaoui, F. Derue, P. Dervan, K. Desch, C. Deterre, P. O. Deviveiros, A. Dewhurst, S. Dhaliwal, A. Di Ciaccio, L. Di Ciaccio, A. Di Domenico, C. Di Donato, A. Di Girolamo, B. Di Girolamo, A. Di Mattia, B. Di Micco, R. Di Nardo, A. Di Simone, R. Di Sipio, D. Di Valentino, C. Diaconu, M. Diamond, F. A. Dias, M. A. Diaz, E. B. Diehl, J. Dietrich, S. Diglio, A. Dimitrievska, J. Dingfelder, P. Dita, S. Dita, F. Dittus, F. Djama, T. Djobava, J. I. Djuvsland, M. A. B. do Vale, D. Dobos, M. Dobre, C. Doglioni, T. Dohmae, J. Dolejsi, Z. Dolezal, B. A. Dolgoshein, M. Donadelli, S. Donati, P. Dondero, J. Donini, J. Dopke, A. Doria, M. T. Dova, A. T. Doyle, E. Drechsler, M. Dris, E. Dubreuil, E. Duchovni, G. Duckeck, O. A. Ducu, D. Duda, A. Dudarev, L. Duflot, L. Duguid, M. Dührssen, M. Dunford, H. Duran Yildiz, M. Düren, A. Durglishvili, D. Duschinger, M. Dyndal, C. Eckardt, K. M. Ecker, R. C. Edgar, W. Edson, N. C. Edwards, W. Ehrenfeld, T. Eifert, G. Eigen, K. Einsweiler, T. Ekelof, M. El Kacimi, M. Ellert, S. Elles, F. Ellinghaus, A. A. Elliot, N. Ellis, J. Elmsheuser, M. Elsing, D. Emeliyanov, Y. Enari, O. C. Endner, M. Endo, J. Erdmann, A. Ereditato, G. Ernis, J. Ernst, M. Ernst, S. Errede, E. Ertel, M. Escalier, H. Esch, C. Escobar, B. Esposito, A. I. Etienvre, E. Etzion, H. Evans, A. Ezhilov, L. Fabbri, G. Facini, R. M. Fakhrutdinov, S. Falciano, R. J. Falla, J. Faltova, Y. Fang, M. Fanti, A. Farbin, A. Farilla, T. Farooque, S. Farrell, S. M. Farrington, P. Farthouat, F. Fassi, P. Fassnacht, D. Fassouliotis, M. Faucci Giannelli, A. Favareto, L. Fayard, P. Federic, O. L. Fedin, W. Fedorko, S. Feigl, L. Feligioni, C. Feng, E. J. Feng, H. Feng, A. B. Fenyuk, L. Feremenga, P. Fernandez Martinez, S. Fernandez Perez, J. Ferrando, A. Ferrari, P. Ferrari, R. Ferrari, D. E. Ferreira de Lima, A. Ferrer, D. Ferrere, C. Ferretti, A. Ferretto Parodi, M. Fiascaris, F. Fiedler, A. Filipčič, M. Filipuzzi, F. Filthaut, M. Fincke-Keeler, K. D. Finelli, M. C. N. Fiolhais, L. Fiorini, A. Firan, A. Fischer, C. Fischer, J. Fischer, W. C. Fisher, E. A. Fitzgerald, N. Flaschel, I. Fleck, P. Fleischmann, S. Fleischmann, G. T. Fletcher, G. Fletcher, R. R. M. Fletcher, T. Flick, A. Floderus, L. R. Flores Castillo, M. J. Flowerdew, A. Formica, A. Forti, D. Fournier, H. Fox, S. Fracchia, P. Francavilla, M. Franchini, D. Francis, L. Franconi, M. Franklin, M. Frate, M. Fraternali, D. Freeborn, S. T. French, F. Friedrich, D. Froidevaux, J. A. Frost, C. Fukunaga, E. Fullana Torregrosa, B. G. Fulsom, T. Fusayasu, J. Fuster, C. Gabaldon, O. Gabizon, A. Gabrielli, A. Gabrielli, G. P. Gach, S. Gadatsch, S. Gadomski, G. Gagliardi, P. Gagnon, C. Galea, B. Galhardo, E. J. Gallas, B. J. Gallop, P. Gallus, G. Galster, K. K. Gan, J. Gao, Y. Gao, Y. S. Gao, F. M. Garay Walls, F. Garberson, C. García, J. E. García Navarro, M. Garcia-Sciveres, R. W. Gardner, N. Garelli, V. Garonne, C. Gatti, A. Gaudiello, G. Gaudio, B. Gaur, L. Gauthier, P. Gauzzi, I. L. Gavrilenko, C. Gay, G. Gaycken, E. N. Gazis, P. Ge, Z. Gecse, C. N. P. Gee, D. A. A. Geerts, Ch. Geich-Gimbel, M. P. Geisler, C. Gemme, M. H. Genest, S. Gentile, M. George, S. George, D. Gerbaudo, A. Gershon, S. Ghasemi, H. Ghazlane, B. Giacobbe, S. Giagu, V. Giangiobbe, P. Giannetti, B. Gibbard, S. M. Gibson, M. Gilchriese, T. P. S. Gillam, D. Gillberg, G. Gilles, D. M. Gingrich, N. Giokaris, M. P. Giordani, F. M. Giorgi, F. M. Giorgi, P. F. Giraud, P. Giromini, D. Giugni, C. Giuliani, M. Giulini, B. K. Gjelsten, S. Gkaitatzis, I. Gkialas, E. L. Gkougkousis, L. K. Gladilin, C. Glasman, J. Glatzer, P. C. F. Glaysher, A. Glazov, M. Goblirsch-Kolb, J. R. Goddard, J. Godlewski, S. Goldfarb, T. Golling, D. Golubkov, A. Gomes, R. Gonçalo, J. Goncalves Pinto Firmino Da Costa, L. Gonella, S. González de la Hoz, G. Gonzalez Parra, S. Gonzalez-Sevilla, L. Goossens, P. A. Gorbounov, H. A. Gordon, I. Gorelov, B. Gorini, E. Gorini, A. Gorišek, E. Gornicki, A. T. Goshaw, C. Gössling, M. I. Gostkin, D. Goujdami, A. G. Goussiou, N. Govender, E. Gozani, H. M. X. Grabas, L. Graber, I. Grabowska-Bold, P. O. J. Gradin, P. Grafström, K-J. Grahn, J. Gramling, E. Gramstad, S. Grancagnolo, V. Grassi, V. Gratchev, H. M. Gray, E. Graziani, Z. D. Greenwood, K. Gregersen, I. M. Gregor, P. Grenier, J. Griffiths, A. A. Grillo, K. Grimm, S. Grinstein, Ph. Gris, J. -F. Grivaz, J. P. Grohs, A. Grohsjean, E. Gross, J. Grosse-Knetter, G. C. Grossi, Z. J. Grout, L. Guan, J. Guenther, F. Guescini, D. Guest, O. Gueta, E. Guido, T. Guillemin, S. Guindon, U. Gul, C. Gumpert, J. Guo, Y. Guo, S. Gupta, G. Gustavino, P. Gutierrez, N. G. Gutierrez Ortiz, C. Gutschow, C. Guyot, C. Gwenlan, C. B. Gwilliam, A. Haas, C. Haber, H. K. Hadavand, N. Haddad, P. Haefner, S. Hageböck, Z. Hajduk, H. Hakobyan, M. Haleem, J. Haley, D. Hall, G. Halladjian, G. D. Hallewell, K. Hamacher, P. Hamal, K. Hamano, M. Hamer, A. Hamilton, G. N. Hamity, P. G. Hamnett, L. Han, K. Hanagaki, K. Hanawa, M. Hance, P. Hanke, R. Hanna, J. B. Hansen, J. D. Hansen, M. C. Hansen, P. H. Hansen, K. Hara, A. S. Hard, T. Harenberg, F. Hariri, S. Harkusha, R. D. Harrington, P. F. Harrison, F. Hartjes, M. Hasegawa, S. Hasegawa, Y. Hasegawa, A. Hasib, S. Hassani, S. Haug, R. Hauser, L. Hauswald, M. Havranek, C. M. Hawkes, R. J. Hawkings, A. D. Hawkins, T. Hayashi, D. Hayden, C. P. Hays, J. M. Hays, H. S. Hayward, S. J. Haywood, S. J. Head, T. Heck, V. Hedberg, L. Heelan, S. Heim, T. Heim, B. Heinemann, L. Heinrich, J. Hejbal, L. Helary, S. Hellman, D. Hellmich, C. Helsens, J. Henderson, R. C. W. Henderson, Y. Heng, C. Hengler, S. Henkelmann, A. Henrichs, A. M. Henriques Correia, S. Henrot-Versille, G. H. Herbert, Y. Hernández Jiménez, R. Herrberg-Schubert, G. Herten, R. Hertenberger, L. Hervas, G. G. Hesketh, N. P. Hessey, J. W. Hetherly, R. Hickling, E. Higón-Rodriguez, E. Hill, J. C. Hill, K. H. Hiller, S. J. Hillier, I. Hinchliffe, E. Hines, R. R. Hinman, M. Hirose, D. Hirschbuehl, J. Hobbs, N. Hod, M. C. Hodgkinson, P. Hodgson, A. Hoecker, M. R. Hoeferkamp, F. Hoenig, M. Hohlfeld, D. Hohn, T. R. Holmes, M. Homann, T. M. Hong, L. Hooft van Huysduynen, W. H. Hopkins, Y. Horii, A. J. Horton, J-Y. Hostachy, S. Hou, A. Hoummada, J. Howard, J. Howarth, M. Hrabovsky, I. Hristova, J. Hrivnac, T. Hryn’ova, A. Hrynevich, C. Hsu, P. J. Hsu, S. -C. Hsu, D. Hu, Q. Hu, X. Hu, Y. Huang, Z. Hubacek, F. Hubaut, F. Huegging, T. B. Huffman, E. W. Hughes, G. Hughes, M. Huhtinen, T. A. Hülsing, N. Huseynov, J. Huston, J. Huth, G. Iacobucci, G. Iakovidis, I. Ibragimov, L. Iconomidou-Fayard, E. Ideal, Z. Idrissi, P. Iengo, O. Igonkina, T. Iizawa, Y. Ikegami, K. Ikematsu, M. Ikeno, Y. Ilchenko, D. Iliadis, N. Ilic, T. Ince, G. Introzzi, P. Ioannou, M. Iodice, K. Iordanidou, V. Ippolito, A. Irles Quiles, C. Isaksson, M. Ishino, M. Ishitsuka, R. Ishmukhametov, C. Issever, S. Istin, J. M. Iturbe Ponce, R. Iuppa, J. Ivarsson, W. Iwanski, H. Iwasaki, J. M. Izen, V. Izzo, S. Jabbar, B. Jackson, M. Jackson, P. Jackson, M. R. Jaekel, V. Jain, K. Jakobs, S. Jakobsen, T. Jakoubek, J. Jakubek, D. O. Jamin, D. K. Jana, E. Jansen, R. Jansky, J. Janssen, M. Janus, G. Jarlskog, N. Javadov, T. Javůrek, L. Jeanty, J. Jejelava, G. -Y. Jeng, D. Jennens, P. Jenni, J. Jentzsch, C. Jeske, S. Jézéquel, H. Ji, J. Jia, Y. Jiang, S. Jiggins, J. Jimenez Pena, S. Jin, A. Jinaru, O. Jinnouchi, M. D. Joergensen, P. Johansson, K. A. Johns, K. Jon-And, G. Jones, R. W. L. Jones, T. J. Jones, J. Jongmanns, P. M. Jorge, K. D. Joshi, J. Jovicevic, X. Ju, C. A. Jung, P. Jussel, A. Juste Rozas, M. Kaci, A. Kaczmarska, M. Kado, H. Kagan, M. Kagan, S. J. Kahn, E. Kajomovitz, C. W. Kalderon, S. Kama, A. Kamenshchikov, N. Kanaya, S. Kaneti, V. A. Kantserov, J. Kanzaki, B. Kaplan, L. S. Kaplan, A. Kapliy, D. Kar, K. Karakostas, A. Karamaoun, N. Karastathis, M. J. Kareem, E. Karentzos, M. Karnevskiy, S. N. Karpov, Z. M. Karpova, K. Karthik, V. Kartvelishvili, A. N. Karyukhin, L. Kashif, R. D. Kass, A. Kastanas, Y. Kataoka, C. Kato, A. Katre, J. Katzy, K. Kawagoe, T. Kawamoto, G. Kawamura, S. Kazama, V. F. Kazanin, R. Keeler, R. Kehoe, J. S. Keller, J. J. Kempster, H. Keoshkerian, O. Kepka, B. P. Kerševan, S. Kersten, R. A. Keyes, F. Khalil-zada, H. Khandanyan, A. Khanov, A. G. Kharlamov, T. J. Khoo, V. Khovanskiy, E. Khramov, J. Khubua, H. Y. Kim, H. Kim, S. H. Kim, Y. K. Kim, N. Kimura, O. M. Kind, B. T. King, M. King, S. B. King, J. Kirk, A. E. Kiryunin, T. Kishimoto, D. Kisielewska, F. Kiss, K. Kiuchi, O. Kivernyk, E. Kladiva, M. H. Klein, M. Klein, U. Klein, K. Kleinknecht, P. Klimek, A. Klimentov, R. Klingenberg, J. A. Klinger, T. Klioutchnikova, E. -E. Kluge, P. Kluit, S. Kluth, J. Knapik, E. Kneringer, E. B. F. G. Knoops, A. Knue, A. Kobayashi, D. Kobayashi, T. Kobayashi, M. Kobel, M. Kocian, P. Kodys, T. Koffas, E. Koffeman, L. A. Kogan, S. Kohlmann, Z. Kohout, T. Kohriki, T. Koi, H. Kolanoski, I. Koletsou, A. A. Komar, Y. Komori, T. Kondo, N. Kondrashova, K. Köneke, A. C. König, T. Kono, R. Konoplich, N. Konstantinidis, R. Kopeliansky, S. Koperny, L. Köpke, A. K. Kopp, K. Korcyl, K. Kordas, A. Korn, A. A. Korol, I. Korolkov, E. V. Korolkova, O. Kortner, S. Kortner, T. Kosek, V. V. Kostyukhin, V. M. Kotov, A. Kotwal, A. Kourkoumeli-Charalampidi, C. Kourkoumelis, V. Kouskoura, A. Koutsman, R. Kowalewski, T. Z. Kowalski, W. Kozanecki, A. S. Kozhin, V. A. Kramarenko, G. Kramberger, D. Krasnopevtsev, M. W. Krasny, A. Krasznahorkay, J. K. Kraus, A. Kravchenko, S. Kreiss, M. Kretz, J. Kretzschmar, K. Kreutzfeldt, P. Krieger, K. Krizka, K. Kroeninger, H. Kroha, J. Kroll, J. Kroseberg, J. Krstic, U. Kruchonak, H. Krüger, N. Krumnack, A. Kruse, M. C. Kruse, M. Kruskal, T. Kubota, H. Kucuk, S. Kuday, S. Kuehn, A. Kugel, F. Kuger, A. Kuhl, T. Kuhl, V. Kukhtin, Y. Kulchitsky, S. Kuleshov, M. Kuna, T. Kunigo, A. Kupco, H. Kurashige, Y. A. Kurochkin, V. Kus, E. S. Kuwertz, M. Kuze, J. Kvita, T. Kwan, D. Kyriazopoulos, A. La Rosa, J. L. La Rosa Navarro, L. La Rotonda, C. Lacasta, F. Lacava, J. Lacey, H. Lacker, D. Lacour, V. R. Lacuesta, E. Ladygin, R. Lafaye, B. Laforge, T. Lagouri, S. Lai, L. Lambourne, S. Lammers, C. L. Lampen, W. Lampl, E. Lançon, U. Landgraf, M. P. J. Landon, V. S. Lang, J. C. Lange, A. J. Lankford, F. Lanni, K. Lantzsch, A. Lanza, S. Laplace, C. Lapoire, J. F. Laporte, T. Lari, F. Lasagni Manghi, M. Lassnig, P. Laurelli, W. Lavrijsen, A. T. Law, P. Laycock, T. Lazovich, O. Le Dortz, E. Le Guirriec, E. Le Menedeu, M. LeBlanc, T. LeCompte, F. Ledroit-Guillon, C. A. Lee, S. C. Lee, L. Lee, G. Lefebvre, M. Lefebvre, F. Legger, C. Leggett, A. Lehan, G. Lehmann Miotto, X. Lei, W. A. Leight, A. Leisos, A. G. Leister, M. A. L. Leite, R. Leitner, D. Lellouch, B. Lemmer, K. J. C. Leney, T. Lenz, B. Lenzi, R. Leone, S. Leone, C. Leonidopoulos, S. Leontsinis, C. Leroy, C. G. Lester, M. Levchenko, J. Levêque, D. Levin, L. J. Levinson, M. Levy, A. Lewis, A. M. Leyko, M. Leyton, B. Li, H. Li, H. L. Li, L. Li, L. Li, S. Li, Y. Li, Z. Liang, H. Liao, B. Liberti, A. Liblong, P. Lichard, K. Lie, J. Liebal, W. Liebig, C. Limbach, A. Limosani, S. C. Lin, T. H. Lin, F. Linde, B. E. Lindquist, J. T. Linnemann, E. Lipeles, A. Lipniacka, M. Lisovyi, T. M. Liss, D. Lissauer, A. Lister, A. M. Litke, B. Liu, D. Liu, H. Liu, J. Liu, J. B. Liu, K. Liu, L. Liu, M. Liu, M. Liu, Y. Liu, M. Livan, A. Lleres, J. Llorente Merino, S. L. Lloyd, F. Lo Sterzo, E. Lobodzinska, P. Loch, W. S. Lockman, F. K. Loebinger, A. E. Loevschall-Jensen, A. Loginov, T. Lohse, K. Lohwasser, M. Lokajicek, B. A. Long, J. D. Long, R. E. Long, K. A. Looper, L. Lopes, D. Lopez Mateos, B. Lopez Paredes, I. Lopez Paz, J. Lorenz, N. Lorenzo Martinez, M. Losada, P. Loscutoff, P. J. Lösel, X. Lou, A. Lounis, J. Love, P. A. Love, N. Lu, H. J. Lubatti, C. Luci, A. Lucotte, F. Luehring, W. Lukas, L. Luminari, O. Lundberg, B. Lund-Jensen, D. Lynn, R. Lysak, E. Lytken, H. Ma, L. L. Ma, G. Maccarrone, A. Macchiolo, C. M. Macdonald, J. Machado Miguens, D. Macina, D. Madaffari, R. Madar, H. J. Maddocks, W. F. Mader, A. Madsen, S. Maeland, T. Maeno, A. Maevskiy, E. Magradze, K. Mahboubi, J. Mahlstedt, C. Maiani, C. Maidantchik, A. A. Maier, T. Maier, A. Maio, S. Majewski, Y. Makida, N. Makovec, B. Malaescu, Pa. Malecki, V. P. Maleev, F. Malek, U. Mallik, D. Malon, C. Malone, S. Maltezos, V. M. Malyshev, S. Malyukov, J. Mamuzic, G. Mancini, B. Mandelli, L. Mandelli, I. Mandić, R. Mandrysch, J. Maneira, A. Manfredini, L. Manhaes de Andrade Filho, J. Manjarres Ramos, A. Mann, P. M. Manning, A. Manousakis-Katsikakis, B. Mansoulie, R. Mantifel, M. Mantoani, L. Mapelli, L. March, G. Marchiori, M. Marcisovsky, C. P. Marino, M. Marjanovic, D. E. Marley, F. Marroquim, S. P. Marsden, Z. Marshall, L. F. Marti, S. Marti-Garcia, B. Martin, T. A. Martin, V. J. Martin, B. Martin dit Latour, M. Martinez, S. Martin-Haugh, V. S. Martoiu, A. C. Martyniuk, M. Marx, F. Marzano, A. Marzin, L. Masetti, T. Mashimo, R. Mashinistov, J. Masik, A. L. Maslennikov, I. Massa, L. Massa, N. Massol, P. Mastrandrea, A. Mastroberardino, T. Masubuchi, P. Mättig, J. Mattmann, J. Maurer, S. J. Maxfield, D. A. Maximov, R. Mazini, S. M. Mazza, L. Mazzaferro, G. Mc Goldrick, S. P. Mc Kee, A. McCarn, R. L. McCarthy, T. G. McCarthy, N. A. McCubbin, K. W. McFarlane, J. A. Mcfayden, G. Mchedlidze, S. J. McMahon, R. A. McPherson, M. Medinnis, S. Meehan, S. Mehlhase, A. Mehta, K. Meier, C. Meineck, B. Meirose, B. R. Mellado Garcia, F. Meloni, A. Mengarelli, S. Menke, E. Meoni, K. M. Mercurio, S. Mergelmeyer, P. Mermod, L. Merola, C. Meroni, F. S. Merritt, A. Messina, J. Metcalfe, A. S. Mete, C. Meyer, C. Meyer, J-P. Meyer, J. Meyer, R. P. Middleton, S. Miglioranzi, L. Mijović, G. Mikenberg, M. Mikestikova, M. Mikuž, M. Milesi, A. Milic, D. W. Miller, C. Mills, A. Milov, D. A. Milstead, A. A. Minaenko, Y. Minami, I. A. Minashvili, A. I. Mincer, B. Mindur, M. Mineev, Y. Ming, L. M. Mir, T. Mitani, J. Mitrevski, V. A. Mitsou, A. Miucci, P. S. Miyagawa, J. U. Mjörnmark, T. Moa, K. Mochizuki, S. Mohapatra, W. Mohr, S. Molander, R. Moles-Valls, K. Mönig, C. Monini, J. Monk, E. Monnier, J. Montejo Berlingen, F. Monticelli, S. Monzani, R. W. Moore, N. Morange, D. Moreno, M. Moreno Llácer, P. Morettini, M. Morgenstern, D. Mori, M. Morii, M. Morinaga, V. Morisbak, S. Moritz, A. K. Morley, G. Mornacchi, J. D. Morris, S. S. Mortensen, A. Morton, L. Morvaj, M. Mosidze, J. Moss, K. Motohashi, R. Mount, E. Mountricha, S. V. Mouraviev, E. J. W. Moyse, S. Muanza, R. D. Mudd, F. Mueller, J. Mueller, R. S. P. Mueller, T. Mueller, D. Muenstermann, P. Mullen, G. A. Mullier, J. A. Murillo Quijada, W. J. Murray, H. Musheghyan, E. Musto, A. G. Myagkov, M. Myska, B. P. Nachman, O. Nackenhorst, J. Nadal, K. Nagai, R. Nagai, Y. Nagai, K. Nagano, A. Nagarkar, Y. Nagasaka, K. Nagata, M. Nagel, E. Nagy, A. M. Nairz, Y. Nakahama, K. Nakamura, T. Nakamura, I. Nakano, H. Namasivayam, R. F. Naranjo Garcia, R. Narayan, T. Naumann, G. Navarro, R. Nayyar, H. A. Neal, P. Yu. Nechaeva, T. J. Neep, P. D. Nef, A. Negri, M. Negrini, S. Nektarijevic, C. Nellist, A. Nelson, S. Nemecek, P. Nemethy, A. A. Nepomuceno, M. Nessi, M. S. Neubauer, M. Neumann, R. M. Neves, P. Nevski, P. R. Newman, D. H. Nguyen, R. B. Nickerson, R. Nicolaidou, B. Nicquevert, J. Nielsen, N. Nikiforou, A. Nikiforov, V. Nikolaenko, I. Nikolic-Audit, K. Nikolopoulos, J. K. Nilsen, P. Nilsson, Y. Ninomiya, A. Nisati, R. Nisius, T. Nobe, M. Nomachi, I. Nomidis, T. Nooney, S. Norberg, M. Nordberg, O. Novgorodova, S. Nowak, M. Nozaki, L. Nozka, K. Ntekas, G. Nunes Hanninger, T. Nunnemann, E. Nurse, F. Nuti, B. J. O’Brien, F. O’grady, D. C. O’Neil, V. O’Shea, F. G. Oakham, H. Oberlack, T. Obermann, J. Ocariz, A. Ochi, I. Ochoa, J. P. Ochoa-Ricoux, S. Oda, S. Odaka, H. Ogren, A. Oh, S. H. Oh, C. C. Ohm, H. Ohman, H. Oide, W. Okamura, H. Okawa, Y. Okumura, T. Okuyama, A. Olariu, S. A. Olivares Pino, D. Oliveira Damazio, E. Oliver Garcia, A. Olszewski, J. Olszowska, A. Onofre, P. U. E. Onyisi, C. J. Oram, M. J. Oreglia, Y. Oren, D. Orestano, N. Orlando, C. Oropeza Barrera, R. S. Orr, B. Osculati, R. Ospanov, G. Otero y Garzon, H. Otono, M. Ouchrif, E. A. Ouellette, F. Ould-Saada, A. Ouraou, K. P. Oussoren, Q. Ouyang, A. Ovcharova, M. Owen, R. E. Owen, V. E. Ozcan, N. Ozturk, K. Pachal, A. Pacheco Pages, C. Padilla Aranda, M. Pagáčová, S. Pagan Griso, E. Paganis, F. Paige, P. Pais, K. Pajchel, G. Palacino, S. Palestini, M. Palka, D. Pallin, A. Palma, Y. B. Pan, E. Panagiotopoulou, C. E. Pandini, J. G. Panduro Vazquez, P. Pani, S. Panitkin, D. Pantea, L. Paolozzi, Th. D. Papadopoulou, K. Papageorgiou, A. Paramonov, D. Paredes Hernandez, M. A. Parker, K. A. Parker, F. Parodi, J. A. Parsons, U. Parzefall, E. Pasqualucci, S. Passaggio, F. Pastore, Fr. Pastore, G. Pásztor, S. Pataraia, N. D. Patel, J. R. Pater, T. Pauly, J. Pearce, B. Pearson, L. E. Pedersen, M. Pedersen, S. Pedraza Lopez, R. Pedro, S. V. Peleganchuk, D. Pelikan, O. Penc, C. Peng, H. Peng, B. Penning, J. Penwell, D. V. Perepelitsa, E. Perez Codina, M. T. Pérez García-Estañ, L. Perini, H. Pernegger, S. Perrella, R. Peschke, V. D. Peshekhonov, K. Peters, R. F. Y. Peters, B. A. Petersen, T. C. Petersen, E. Petit, A. Petridis, C. Petridou, P. Petroff, E. Petrolo, F. Petrucci, N. E. Pettersson, R. Pezoa, P. W. Phillips, G. Piacquadio, E. Pianori, A. Picazio, E. Piccaro, M. Piccinini, M. A. Pickering, R. Piegaia, D. T. Pignotti, J. E. Pilcher, A. D. Pilkington, J. Pina, M. Pinamonti, J. L. Pinfold, A. Pingel, B. Pinto, S. Pires, H. Pirumov, M. Pitt, C. Pizio, L. Plazak, M. -A. Pleier, V. Pleskot, E. Plotnikova, P. Plucinski, D. Pluth, R. Poettgen, L. Poggioli, D. Pohl, G. Polesello, A. Poley, A. Policicchio, R. Polifka, A. Polini, C. S. Pollard, V. Polychronakos, K. Pommès, L. Pontecorvo, B. G. Pope, G. A. Popeneciu, D. S. Popovic, A. Poppleton, S. Pospisil, K. Potamianos, I. N. Potrap, C. J. Potter, C. T. Potter, G. Poulard, J. Poveda, V. Pozdnyakov, P. Pralavorio, A. Pranko, S. Prasad, S. Prell, D. Price, L. E. Price, M. Primavera, S. Prince, M. Proissl, K. Prokofiev, F. Prokoshin, E. Protopapadaki, S. Protopopescu, J. Proudfoot, M. Przybycien, E. Ptacek, D. Puddu, E. Pueschel, D. Puldon, M. Purohit, P. Puzo, J. Qian, G. Qin, Y. Qin, A. Quadt, D. R. Quarrie, W. B. Quayle, M. Queitsch-Maitland, D. Quilty, S. Raddum, V. Radeka, V. Radescu, S. K. Radhakrishnan, P. Radloff, P. Rados, F. Ragusa, G. Rahal, S. Rajagopalan, M. Rammensee, C. Rangel-Smith, F. Rauscher, S. Rave, T. Ravenscroft, M. Raymond, A. L. Read, N. P. Readioff, D. M. Rebuzzi, A. Redelbach, G. Redlinger, R. Reece, K. Reeves, L. Rehnisch, J. Reichert, H. Reisin, M. Relich, C. Rembser, H. Ren, A. Renaud, M. Rescigno, S. Resconi, O. L. Rezanova, P. Reznicek, R. Rezvani, R. Richter, S. Richter, E. Richter-Was, O. Ricken, M. Ridel, P. Rieck, C. J. Riegel, J. Rieger, M. Rijssenbeek, A. Rimoldi, L. Rinaldi, B. Ristić, E. Ritsch, I. Riu, F. Rizatdinova, E. Rizvi, S. H. Robertson, A. Robichaud-Veronneau, D. Robinson, J. E. M. Robinson, A. Robson, C. Roda, S. Roe, O. Røhne, S. Rolli, A. Romaniouk, M. Romano, S. M. Romano Saez, E. Romero Adam, N. Rompotis, M. Ronzani, L. Roos, E. Ros, S. Rosati, K. Rosbach, P. Rose, P. L. Rosendahl, O. Rosenthal, V. Rossetti, E. Rossi, L. P. Rossi, R. Rosten, M. Rotaru, I. Roth, J. Rothberg, D. Rousseau, C. R. Royon, A. Rozanov, Y. Rozen, X. Ruan, F. Rubbo, I. Rubinskiy, V. I. Rud, C. Rudolph, M. S. Rudolph, F. Rühr, A. Ruiz-Martinez, Z. Rurikova, N. A. Rusakovich, A. Ruschke, H. L. Russell, J. P. Rutherfoord, N. Ruthmann, Y. F. Ryabov, M. Rybar, G. Rybkin, N. C. Ryder, A. F. Saavedra, G. Sabato, S. Sacerdoti, A. Saddique, H. F-W. Sadrozinski, R. Sadykov, F. Safai Tehrani, M. Sahinsoy, M. Saimpert, T. Saito, H. Sakamoto, Y. Sakurai, G. Salamanna, A. Salamon, M. Saleem, D. Salek, P. H. Sales De Bruin, D. Salihagic, A. Salnikov, J. Salt, D. Salvatore, F. Salvatore, A. Salvucci, A. Salzburger, D. Sammel, D. Sampsonidis, A. Sanchez, J. Sánchez, V. Sanchez Martinez, H. Sandaker, R. L. Sandbach, H. G. Sander, M. P. Sanders, M. Sandhoff, C. Sandoval, R. Sandstroem, D. P. C. Sankey, M. Sannino, A. Sansoni, C. Santoni, R. Santonico, H. Santos, I. Santoyo Castillo, K. Sapp, A. Sapronov, J. G. Saraiva, B. Sarrazin, O. Sasaki, Y. Sasaki, K. Sato, G. Sauvage, E. Sauvan, G. Savage, P. Savard, C. Sawyer, L. Sawyer, J. Saxon, C. Sbarra, A. Sbrizzi, T. Scanlon, D. A. Scannicchio, M. Scarcella, V. Scarfone, J. Schaarschmidt, P. Schacht, D. Schaefer, R. Schaefer, J. Schaeffer, S. Schaepe, S. Schaetzel, U. Schäfer, A. C. Schaffer, D. Schaile, R. D. Schamberger, V. Scharf, V. A. Schegelsky, D. Scheirich, M. Schernau, C. Schiavi, C. Schillo, M. Schioppa, S. Schlenker, E. Schmidt, K. Schmieden, C. Schmitt, S. Schmitt, S. Schmitt, B. Schneider, Y. J. Schnellbach, U. Schnoor, L. Schoeffel, A. Schoening, B. D. Schoenrock, E. Schopf, A. L. S. Schorlemmer, M. Schott, D. Schouten, J. Schovancova, S. Schramm, M. Schreyer, C. Schroeder, N. Schuh, M. J. Schultens, H.-C. Schultz-Coulon, H. Schulz, M. Schumacher, B. A. Schumm, Ph. Schune, C. Schwanenberger, A. Schwartzman, T. A. Schwarz, Ph. Schwegler, H. Schweiger, Ph. Schwemling, R. Schwienhorst, J. Schwindling, T. Schwindt, F. G. Sciacca, E. Scifo, G. Sciolla, F. Scuri, F. Scutti, J. Searcy, G. Sedov, E. Sedykh, P. Seema, S. C. Seidel, A. Seiden, F. Seifert, J. M. Seixas, G. Sekhniaidze, K. Sekhon, S. J. Sekula, D. M. Seliverstov, N. Semprini-Cesari, C. Serfon, L. Serin, L. Serkin, T. Serre, M. Sessa, R. Seuster, H. Severini, T. Sfiligoj, F. Sforza, A. Sfyrla, E. Shabalina, M. Shamim, L. Y. Shan, R. Shang, J. T. Shank, M. Shapiro, P. B. Shatalov, K. Shaw, S. M. Shaw, A. Shcherbakova, C. Y. Shehu, P. Sherwood, L. Shi, S. Shimizu, C. O. Shimmin, M. Shimojima, M. Shiyakova, A. Shmeleva, D. Shoaleh Saadi, M. J. Shochet, S. Shojaii, S. Shrestha, E. Shulga, M. A. Shupe, S. Shushkevich, P. Sicho, P. E. Sidebo, O. Sidiropoulou, D. Sidorov, A. Sidoti, F. Siegert, Dj. Sijacki, J. Silva, Y. Silver, S. B. Silverstein, V. Simak, O. Simard, Lj. Simic, S. Simion, E. Simioni, B. Simmons, D. Simon, R. Simoniello, P. Sinervo, N. B. Sinev, M. Sioli, G. Siragusa, A. N. Sisakyan, S. Yu. Sivoklokov, J. Sjölin, T. B. Sjursen, M. B. Skinner, H. P. Skottowe, P. Skubic, M. Slater, T. Slavicek, M. Slawinska, K. Sliwa, V. Smakhtin, B. H. Smart, L. Smestad, S. Yu. Smirnov, Y. Smirnov, L. N. Smirnova, O. Smirnova, M. N. K. Smith, R. W. Smith, M. Smizanska, K. Smolek, A. A. Snesarev, G. Snidero, S. Snyder, R. Sobie, F. Socher, A. Soffer, D. A. Soh, C. A. Solans, M. Solar, J. Solc, E. Yu. Soldatov, U. Soldevila, A. A. Solodkov, A. Soloshenko, O. V. Solovyanov, V. Solovyev, P. Sommer, H. Y. Song, N. Soni, A. Sood, A. Sopczak, B. Sopko, V. Sopko, V. Sorin, D. Sosa, M. Sosebee, C. L. Sotiropoulou, R. Soualah, A. M. Soukharev, D. South, B. C. Sowden, S. Spagnolo, M. Spalla, F. Spanò, W. R. Spearman, D. Sperlich, F. Spettel, R. Spighi, G. Spigo, L. A. Spiller, M. Spousta, T. Spreitzer, R. D. St. Denis, S. Staerz, J. Stahlman, R. Stamen, S. Stamm, E. Stanecka, C. Stanescu, M. Stanescu-Bellu, M. M. Stanitzki, S. Stapnes, E. A. Starchenko, J. Stark, P. Staroba, P. Starovoitov, R. Staszewski, P. Stavina, P. Steinberg, B. Stelzer, H. J. Stelzer, O. Stelzer-Chilton, H. Stenzel, G. A. Stewart, J. A. Stillings, M. C. Stockton, M. Stoebe, G. Stoicea, P. Stolte, S. Stonjek, A. R. Stradling, A. Straessner, M. E. Stramaglia, J. Strandberg, S. Strandberg, A. Strandlie, E. Strauss, M. Strauss, P. Strizenec, R. Ströhmer, D. M. Strom, R. Stroynowski, A. Strubig, S. A. Stucci, B. Stugu, N. A. Styles, D. Su, J. Su, R. Subramaniam, A. Succurro, Y. Sugaya, C. Suhr, M. Suk, V. V. Sulin, S. Sultansoy, T. Sumida, S. Sun, X. Sun, J. E. Sundermann, K. Suruliz, G. Susinno, M. R. Sutton, S. Suzuki, M. Svatos, S. Swedish, M. Swiatlowski, I. Sykora, T. Sykora, D. Ta, C. Taccini, K. Tackmann, J. Taenzer, A. Taffard, R. Tafirout, N. Taiblum, H. Takai, R. Takashima, H. Takeda, T. Takeshita, Y. Takubo, M. Talby, A. A. Talyshev, J. Y. C. Tam, K. G. Tan, J. Tanaka, R. Tanaka, S. Tanaka, B. B. Tannenwald, N. Tannoury, S. Tapprogge, S. Tarem, F. Tarrade, G. F. Tartarelli, P. Tas, M. Tasevsky, T. Tashiro, E. Tassi, A. Tavares Delgado, Y. Tayalati, F. E. Taylor, G. N. Taylor, W. Taylor, F. A. Teischinger, M. Teixeira Dias Castanheira, P. Teixeira-Dias, K. K. Temming, H. Ten Kate, P. K. Teng, J. J. Teoh, F. Tepel, S. Terada, K. Terashi, J. Terron, S. Terzo, M. Testa, R. J. Teuscher, T. Theveneaux-Pelzer, J. P. Thomas, J. Thomas-Wilsker, E. N. Thompson, P. D. Thompson, R. J. Thompson, A. S. Thompson, L. A. Thomsen, E. Thomson, M. Thomson, R. P. Thun, M. J. Tibbetts, R. E. Ticse Torres, V. O. Tikhomirov, Yu. A. Tikhonov, S. Timoshenko, E. Tiouchichine, P. Tipton, S. Tisserant, K. Todome, T. Todorov, S. Todorova-Nova, J. Tojo, S. Tokár, K. Tokushuku, K. Tollefson, E. Tolley, L. Tomlinson, M. Tomoto, L. Tompkins, K. Toms, E. Torrence, H. Torres, E. Torró Pastor, J. Toth, F. Touchard, D. R. Tovey, T. Trefzger, L. Tremblet, A. Tricoli, I. M. Trigger, S. Trincaz-Duvoid, M. F. Tripiana, W. Trischuk, B. Trocmé, C. Troncon, M. Trottier-McDonald, M. Trovatelli, P. True, L. Truong, M. Trzebinski, A. Trzupek, C. Tsarouchas, J. C-L. Tseng, P. V. Tsiareshka, D. Tsionou, G. Tsipolitis, N. Tsirintanis, S. Tsiskaridze, V. Tsiskaridze, E. G. Tskhadadze, I. I. Tsukerman, V. Tsulaia, S. Tsuno, D. Tsybychev, A. Tudorache, V. Tudorache, A. N. Tuna, S. A. Tupputi, S. Turchikhin, D. Turecek, R. Turra, A. J. Turvey, P. M. Tuts, A. Tykhonov, M. Tylmad, M. Tyndel, I. Ueda, R. Ueno, M. Ughetto, M. Ugland, M. Uhlenbrock, F. Ukegawa, G. Unal, A. Undrus, G. Unel, F. C. Ungaro, Y. Unno, C. Unverdorben, J. Urban, P. Urquijo, P. Urrejola, G. Usai, A. Usanova, L. Vacavant, V. Vacek, B. Vachon, C. Valderanis, N. Valencic, S. Valentinetti, A. Valero, L. Valery, S. Valkar, E. Valladolid Gallego, S. Vallecorsa, J. A. Valls Ferrer, W. Van Den Wollenberg, P. C. Van Der Deijl, R. van der Geer, H. van der Graaf, R. Van Der Leeuw, N. van Eldik, P. van Gemmeren, J. Van Nieuwkoop, I. van Vulpen, M. C. van Woerden, M. Vanadia, W. Vandelli, R. Vanguri, A. Vaniachine, F. Vannucci, G. Vardanyan, R. Vari, E. W. Varnes, T. Varol, D. Varouchas, A. Vartapetian, K. E. Varvell, V. I. Vassilakopoulos, F. Vazeille, T. Vazquez Schroeder, J. Veatch, L. M. Veloce, F. Veloso, T. Velz, S. Veneziano, A. Ventura, D. Ventura, M. Venturi, N. Venturi, A. Venturini, V. Vercesi, M. Verducci, W. Verkerke, J. C. Vermeulen, A. Vest, M. C. Vetterli, O. Viazlo, I. Vichou, T. Vickey, O. E. Vickey Boeriu, G. H. A. Viehhauser, S. Viel, R. Vigne, M. Villa, M. Villaplana Perez, E. Vilucchi, M. G. Vincter, V. B. Vinogradov, I. Vivarelli, F. Vives Vaque, S. Vlachos, D. Vladoiu, M. Vlasak, M. Vogel, P. Vokac, G. Volpi, M. Volpi, H. von der Schmitt, H. von Radziewski, E. von Toerne, V. Vorobel, K. Vorobev, M. Vos, R. Voss, J. H. Vossebeld, N. Vranjes, M. Vranjes Milosavljevic, V. Vrba, M. Vreeswijk, R. Vuillermet, I. Vukotic, Z. Vykydal, P. Wagner, W. Wagner, H. Wahlberg, S. Wahrmund, J. Wakabayashi, J. Walder, R. Walker, W. Walkowiak, C. Wang, F. Wang, H. Wang, H. Wang, J. Wang, J. Wang, K. Wang, R. Wang, S. M. Wang, T. Wang, T. Wang, X. Wang, C. Wanotayaroj, A. Warburton, C. P. Ward, D. R. Wardrope, M. Warsinsky, A. Washbrook, C. Wasicki, P. M. Watkins, A. T. Watson, I. J. Watson, M. F. Watson, G. Watts, S. Watts, B. M. Waugh, S. Webb, M. S. Weber, S. W. Weber, J. S. Webster, A. R. Weidberg, B. Weinert, J. Weingarten, C. Weiser, H. Weits, P. S. Wells, T. Wenaus, T. Wengler, S. Wenig, N. Wermes, M. Werner, P. Werner, M. Wessels, J. Wetter, K. Whalen, A. M. Wharton, A. White, M. J. White, R. White, S. White, D. Whiteson, F. J. Wickens, W. Wiedenmann, M. Wielers, P. Wienemann, C. Wiglesworth, L. A. M. Wiik-Fuchs, A. Wildauer, H. G. Wilkens, H. H. Williams, S. Williams, C. Willis, S. Willocq, A. Wilson, J. A. Wilson, I. Wingerter-Seez, F. Winklmeier, B. T. Winter, M. Wittgen, J. Wittkowski, S. J. Wollstadt, M. W. Wolter, H. Wolters, B. K. Wosiek, J. Wotschack, M. J. Woudstra, K. W. Wozniak, M. Wu, M. Wu, S. L. Wu, X. Wu, Y. Wu, T. R. Wyatt, B. M. Wynne, S. Xella, D. Xu, L. Xu, B. Yabsley, S. Yacoob, R. Yakabe, M. Yamada, Y. Yamaguchi, A. Yamamoto, S. Yamamoto, T. Yamanaka, K. Yamauchi, Y. Yamazaki, Z. Yan, H. Yang, H. Yang, Y. Yang, W-M. Yao, Y. Yasu, E. Yatsenko, K. H. Yau Wong, J. Ye, S. Ye, I. Yeletskikh, A. L. Yen, E. Yildirim, K. Yorita, R. Yoshida, K. Yoshihara, C. Young, C. J. S. Young, S. Youssef, D. R. Yu, J. Yu, J. M. Yu, J. Yu, L. Yuan, S. P. Y. Yuen, A. Yurkewicz, I. Yusuff, B. Zabinski, R. Zaidan, A. M. Zaitsev, J. Zalieckas, A. Zaman, S. Zambito, L. Zanello, D. Zanzi, C. Zeitnitz, M. Zeman, A. Zemla, K. Zengel, O. Zenin, T. Ženiš, D. Zerwas, D. Zhang, F. Zhang, H. Zhang, J. Zhang, L. Zhang, R. Zhang, X. Zhang, Z. Zhang, X. Zhao, Y. Zhao, Z. Zhao, A. Zhemchugov, J. Zhong, B. Zhou, C. Zhou, L. Zhou, L. Zhou, N. Zhou, C. G. Zhu, H. Zhu, J. Zhu, Y. Zhu, X. Zhuang, K. Zhukov, A. Zibell, D. Zieminska, N. I. Zimine, C. Zimmermann, S. Zimmermann, Z. Zinonos, M. Zinser, M. Ziolkowski, L. Živković, G. Zobernig, A. Zoccoli, M. zur Nedden, G. Zurzolo, L. Zwalinski, ATLAS Collaboration

**Affiliations:** 1 Department of Physics, University of Adelaide, Adelaide, Australia; 2 Physics Department, SUNY Albany, Albany, NY USA; 3 Department of Physics, University of Alberta, Edmonton, AB Canada; 4 Department of Physics, Ankara University, Ankara, Turkey; 5 Istanbul Aydin University, Istanbul, Turkey; 6 Division of Physics, TOBB University of Economics and Technology, Ankara, Turkey; 7 LAPP, CNRS/IN2P3 and Université Savoie Mont Blanc, Annecy-le-Vieux, France; 8 High Energy Physics Division, Argonne National Laboratory, Argonne, IL USA; 9 Department of Physics, University of Arizona, Tucson, AZ USA; 10 Department of Physics, The University of Texas at Arlington, Arlington, TX USA; 11 Physics Department, University of Athens, Athens, Greece; 12 Physics Department, National Technical University of Athens, Zografou, Greece; 13 Institute of Physics, Azerbaijan Academy of Sciences, Baku, Azerbaijan; 14 Departament de Física de la Universitat Autònoma de Barcelona, Institut de Física d’Altes Energies, Barcelona, Spain; 15 Institute of Physics, University of Belgrade, Belgrade, Serbia; 16 Department for Physics and Technology, University of Bergen, Bergen, Norway; 17 Physics Division, Lawrence Berkeley National Laboratory, University of California, Berkeley, CA USA; 18 Department of Physics, Humboldt University, Berlin, Germany; 19 Albert Einstein Center for Fundamental Physics and Laboratory for High Energy Physics, University of Bern, Bern, Switzerland; 20 School of Physics and Astronomy, University of Birmingham, Birmingham, UK; 21 Department of Physics, Bogazici University, Istanbul, Turkey; 22 Department of Physics Engineering, Gaziantep University, Gaziantep, Turkey; 23 Department of Physics, Dogus University, Istanbul, Turkey; 24 INFN Sezione di Bologna, Bologna, Italy; 25 Dipartimento di Fisica e Astronomia, Università di Bologna, Bologna, Italy; 26 Physikalisches Institut, University of Bonn, Bonn, Germany; 27 Department of Physics, Boston University, Boston, MA USA; 28 Department of Physics, Brandeis University, Waltham, MA USA; 29 Universidade Federal do Rio De Janeiro COPPE/EE/IF, Rio de Janeiro, Brazil; 30 Electrical Circuits Department, Federal University of Juiz de Fora (UFJF), Juiz de Fora, Brazil; 31 Federal University of Sao Joao del Rei (UFSJ), Sao Joao del Rei, Brazil; 32 Instituto de Fisica, Universidade de Sao Paulo, Sao Paulo, Brazil; 33 Physics Department, Brookhaven National Laboratory, Upton, NY USA; 34 National Institute of Physics and Nuclear Engineering, Bucharest, Romania; 35 Physics Department, National Institute for Research and Development of Isotopic and Molecular Technologies, Cluj Napoca, Romania; 36 University Politehnica Bucharest, Bucharest, Romania; 37 West University in Timisoara, Timisoara, Romania; 38 Departamento de Física, Universidad de Buenos Aires, Buenos Aires, Argentina; 39 Cavendish Laboratory, University of Cambridge, Cambridge, UK; 40 Department of Physics, Carleton University, Ottawa, ON Canada; 41 CERN, Geneva, Switzerland; 42 Enrico Fermi Institute, University of Chicago, Chicago, IL USA; 43 Departamento de Física, Pontificia Universidad Católica de Chile, Santiago, Chile; 44 Departamento de Física, Universidad Técnica Federico Santa María, Valparaiso, Chile; 45 Institute of High Energy Physics, Chinese Academy of Sciences, Beijing, China; 46 Department of Modern Physics, University of Science and Technology of China, Hefei, Anhui China; 47 Department of Physics, Nanjing University, Jiangsu, China; 48 School of Physics, Shandong University, Shandong, China; 49 Department of Physics and Astronomy, Shanghai Key Laboratory for Particle Physics and Cosmology, Shanghai Jiao Tong University, Shanghai, China; 50 Physics Department, Tsinghua University, Beijing, 100084 China; 51 Laboratoire de Physique Corpusculaire, Clermont Université and Université Blaise Pascal and CNRS/IN2P3, Clermont-Ferrand, France; 52 Nevis Laboratory, Columbia University, Irvington, NY USA; 53 Niels Bohr Institute, University of Copenhagen, Copenhagen, Denmark; 54 INFN Gruppo Collegato di Cosenza, Laboratori Nazionali di Frascati, Rende, Italy; 55 Dipartimento di Fisica, Università della Calabria, Rende, Italy; 56 Faculty of Physics and Applied Computer Science, AGH University of Science and Technology, Kraków, Poland; 57 Marian Smoluchowski Institute of Physics, Jagiellonian University, Kraków, Poland; 58 Institute of Nuclear Physics, Polish Academy of Sciences, Kraków, Poland; 59 Physics Department, Southern Methodist University, Dallas, TX USA; 60 Physics Department, University of Texas at Dallas, Richardson, TX USA; 61 DESY, Hamburg and Zeuthen, Germany; 62 Institut für Experimentelle Physik IV, Technische Universität Dortmund, Dortmund, Germany; 63 Institut für Kern-und Teilchenphysik, Technische Universität Dresden, Dresden, Germany; 64 Department of Physics, Duke University, Durham, NC USA; 65 SUPA-School of Physics and Astronomy, University of Edinburgh, Edinburgh, UK; 66 INFN Laboratori Nazionali di Frascati, Frascati, Italy; 67 Fakultät für Mathematik und Physik, Albert-Ludwigs-Universität, Freiburg, Germany; 68 Section de Physique, Université de Genève, Geneva, Switzerland; 69 INFN Sezione di Genova, Genoa, Italy; 70 Dipartimento di Fisica, Università di Genova, Genoa, Italy; 71 E. Andronikashvili Institute of Physics, Iv. Javakhishvili Tbilisi State University, Tbilisi, Georgia; 72 High Energy Physics Institute, Tbilisi State University, Tbilisi, Georgia; 73 II Physikalisches Institut, Justus-Liebig-Universität Giessen, Giessen, Germany; 74 SUPA-School of Physics and Astronomy, University of Glasgow, Glasgow, UK; 75 II Physikalisches Institut, Georg-August-Universität, Göttingen, Germany; 76 Laboratoire de Physique Subatomique et de Cosmologie, Université Grenoble-Alpes, CNRS/IN2P3, Grenoble, France; 77 Department of Physics, Hampton University, Hampton, VA USA; 78 Laboratory for Particle Physics and Cosmology, Harvard University, Cambridge, MA USA; 79 Kirchhoff-Institut für Physik, Ruprecht-Karls-Universität Heidelberg, Heidelberg, Germany; 80 Physikalisches Institut, Ruprecht-Karls-Universität Heidelberg, Heidelberg, Germany; 81 ZITI Institut für technische Informatik, Ruprecht-Karls-Universität Heidelberg, Mannheim, Germany; 82 Faculty of Applied Information Science, Hiroshima Institute of Technology, Hiroshima, Japan; 83 Department of Physics, The Chinese University of Hong Kong, Shatin, N.T. Hong Kong; 84 Department of Physics, The University of Hong Kong, Pokfulam, Hong Kong; 85 Department of Physics, The Hong Kong University of Science and Technology, Clear Water Bay, Kowloon, Hong Kong, China; 86 Department of Physics, Indiana University, Bloomington, IN USA; 87 Institut für Astro- und Teilchenphysik, Leopold-Franzens-Universität, Innsbruck, Austria; 88 University of Iowa, Iowa City, IA USA; 89 Department of Physics and Astronomy, Iowa State University, Ames, IA USA; 90 Joint Institute for Nuclear Research, JINR Dubna, Dubna, Russia; 91 KEK, High Energy Accelerator Research Organization, Tsukuba, Japan; 92 Graduate School of Science, Kobe University, Kobe, Japan; 93 Faculty of Science, Kyoto University, Kyoto, Japan; 94 Kyoto University of Education, Kyoto, Japan; 95 Department of Physics, Kyushu University, Fukuoka, Japan; 96 Instituto de Física La Plata, Universidad Nacional de La Plata and CONICET, La Plata, Argentina; 97 Physics Department, Lancaster University, Lancaster, UK; 98 INFN Sezione di Lecce, Lecce, Italy; 99 Dipartimento di Matematica e Fisica, Università del Salento, Lecce, Italy; 100 Oliver Lodge Laboratory, University of Liverpool, Liverpool, UK; 101 Department of Physics, Jožef Stefan Institute and University of Ljubljana, Ljubljana, Slovenia; 102 School of Physics and Astronomy, Queen Mary University of London, London, UK; 103 Department of Physics, Royal Holloway University of London, Surrey, UK; 104 Department of Physics and Astronomy, University College London, London, UK; 105 Louisiana Tech University, Ruston, LA USA; 106 Laboratoire de Physique Nucléaire et de Hautes Energies, UPMC and Université Paris-Diderot and CNRS/IN2P3, Paris, France; 107 Fysiska institutionen, Lunds universitet, Lund, Sweden; 108 Departamento de Fisica Teorica C-15, Universidad Autonoma de Madrid, Madrid, Spain; 109 Institut für Physik, Universität Mainz, Mainz, Germany; 110 School of Physics and Astronomy, University of Manchester, Manchester, UK; 111 CPPM, Aix-Marseille Université and CNRS/IN2P3, Marseille, France; 112 Department of Physics, University of Massachusetts, Amherst, MA USA; 113 Department of Physics, McGill University, Montreal, QC Canada; 114 School of Physics, University of Melbourne, Melbourne, VIC Australia; 115 Department of Physics, The University of Michigan, Ann Arbor, MI USA; 116 Department of Physics and Astronomy, Michigan State University, East Lansing, MI USA; 117 INFN Sezione di Milano, Milan, Italy; 118 Dipartimento di Fisica, Università di Milano, Milan, Italy; 119 B.I. Stepanov Institute of Physics, National Academy of Sciences of Belarus, Minsk, Republic of Belarus; 120 National Scientific and Educational Centre for Particle and High Energy Physics, Minsk, Republic of Belarus; 121 Department of Physics, Massachusetts Institute of Technology, Cambridge, MA USA; 122 Group of Particle Physics, University of Montreal, Montreal, QC Canada; 123 P.N. Lebedev Physical Institute of the Russian, Academy of Sciences, Moscow, Russia; 124 Institute for Theoretical and Experimental Physics (ITEP), Moscow, Russia; 125 National Research Nuclear University MEPhI, Moscow, Russia; 126 D.V. Skobeltsyn Institute of Nuclear Physics, M.V. Lomonosov Moscow State University, Moscow, Russia; 127 Fakultät für Physik, Ludwig-Maximilians-Universität München, Munich, Germany; 128 Max-Planck-Institut für Physik (Werner-Heisenberg-Institut), Munich, Germany; 129 Nagasaki Institute of Applied Science, Nagasaki, Japan; 130 Graduate School of Science and Kobayashi-Maskawa Institute, Nagoya University, Nagoya, Japan; 131 INFN Sezione di Napoli, Naples, Italy; 132 Dipartimento di Fisica, Università di Napoli, Naples, Italy; 133 Department of Physics and Astronomy, University of New Mexico, Albuquerque, NM USA; 134 Institute for Mathematics, Astrophysics and Particle Physics, Radboud University Nijmegen/Nikhef, Nijmegen, The Netherlands; 135 Nikhef National Institute for Subatomic Physics and University of Amsterdam, Amsterdam, The Netherlands; 136 Department of Physics, Northern Illinois University, DeKalb, IL USA; 137 Budker Institute of Nuclear Physics, SB RAS, Novosibirsk, Russia; 138 Department of Physics, New York University, New York, NY USA; 139 Ohio State University, Columbus, OH USA; 140 Faculty of Science, Okayama University, Okayama, Japan; 141 Homer L. Dodge Department of Physics and Astronomy, University of Oklahoma, Norman, OK USA; 142 Department of Physics, Oklahoma State University, Stillwater, OK USA; 143 Palacký University, RCPTM, Olomouc, Czech Republic; 144 Center for High Energy Physics, University of Oregon, Eugene, OR USA; 145 LAL, Université Paris-Sub and CNRS/IN2P3, Orsay, France; 146 Graduate School of Science, Osaka University, Osaka, Japan; 147 Department of Physics, University of Oslo, Oslo, Norway; 148 Department of Physics, Oxford University, Oxford, UK; 149 INFN Sezione di Pavia, Pavia, Italy; 150 Dipartimento di Fisica, Università di Pavia, Pavia, Italy; 151 Department of Physics, University of Pennsylvania, Philadelphia, PA USA; 152 National Research Centre “Kurchatov Institute” B.P. Konstantinov Petersburg Nuclear Physics Institute, St. Petersburg, Russia; 153 INFN Sezione di Pisa, Pisa, Italy; 154 Dipartimento di Fisica E. Fermi, Università di Pisa, Pisa, Italy; 155 Department of Physics and Astronomy, University of Pittsburgh, Pittsburgh, PA USA; 156 Laboratório de Instrumentação e Física Experimental de Partículas-LIP, Lisbon, Portugal; 157 Faculdade de Ciências, Universidade de Lisboa, Lisbon, Portugal; 158 Department of Physics, University of Coimbra, Coimbra, Portugal; 159 Centro de Física Nuclear da Universidade de Lisboa, Lisbon, Portugal; 160 Departamento de Fisica, Universidade do Minho, Braga, Portugal; 161 Departamento de Fisica Teorica y del Cosmos and CAFPE, Universidad de Granada, Granada, Spain; 162 Dep Fisica and CEFITEC of Faculdade de Ciencias e Tecnologia, Universidade Nova de Lisboa, Caparica, Portugal; 163 Institute of Physics, Academy of Sciences of the Czech Republic, Prague, Czech Republic; 164 Czech Technical University in Prague, Prague, Czech Republic; 165 Faculty of Mathematics and Physics, Charles University in Prague, Prague, Czech Republic; 166 State Research Center Institute for High Energy Physics, Protvino, Russia; 167 Particle Physics Department, Rutherford Appleton Laboratory, Didcot, UK; 168 INFN Sezione di Roma, Rome, Italy; 169 Dipartimento di Fisica, Sapienza Università di Roma, Rome, Italy; 170 INFN Sezione di Roma Tor Vergata, Rome, Italy; 171 Dipartimento di Fisica, Università di Roma Tor Vergata, Rome, Italy; 172 INFN Sezione di Roma Tre, Rome, Italy; 173 Dipartimento di Matematica e Fisica, Università Roma Tre, Rome, Italy; 174 Faculté des Sciences Ain Chock, Réseau Universitaire de Physique des Hautes Energies-Université Hassan II, Casablanca, Morocco; 175 Centre National de l’Energie des Sciences Techniques Nucleaires, Rabat, Morocco; 176 Faculté des Sciences Semlalia, Université Cadi Ayyad, LPHEA-Marrakech, Marrakesh, Morocco; 177 Faculté des Sciences, Université Mohamed Premier and LPTPM, Oujda, Morocco; 178 Faculté des , Sciences, Université Mohammed V, Rabat, Morocco; 179 DSM/IRFU (Institut de Recherches sur les Lois Fondamentales de l’Univers), CEA Saclay (Commissariat à l’Energie Atomique et aux Energies Alternatives), Gif-sur-Yvette, France; 180 Santa Cruz Institute for Particle Physics, University of California Santa Cruz, Santa Cruz, CA USA; 181 Department of Physics, University of Washington, Seattle, WA USA; 182 Department of Physics and Astronomy, University of Sheffield, Sheffield, UK; 183 Department of Physics, Shinshu University, Nagano, Japan; 184 Fachbereich Physik, Universität Siegen, Siegen, Germany; 185 Department of Physics, Simon Fraser University, Burnaby, BC Canada; 186 SLAC National Accelerator Laboratory, Stanford, CA USA; 187 Faculty of Mathematics, Physics and Informatics, Comenius University, Bratislava, Slovakia; 188 Department of Subnuclear Physics, Institute of Experimental Physics of the Slovak Academy of Sciences, Kosice, Slovak Republic; 189 Department of Physics, University of Cape Town, Cape Town, South Africa; 190 Department of Physics, University of Johannesburg, Johannesburg, South Africa; 191 School of Physics, University of the Witwatersrand, Johannesburg, South Africa; 192 Department of Physics, Stockholm University, Stockholm, Sweden; 193 The Oskar Klein Centre, Stockholm, Sweden; 194 Physics Department, Royal Institute of Technology, Stockholm, Sweden; 195 Departments of Physics and Astronomy and Chemistry, Stony Brook University, Stony Brook, NY USA; 196 Department of Physics and Astronomy, University of Sussex, Brighton, UK; 197 School of Physics, University of Sydney, Sydney, Australia; 198 Institute of Physics, Academia Sinica, Taipei, Taiwan; 199 Department of Physics, Technion: Israel Institute of Technology, Haifa, Israel; 200 Raymond and Beverly Sackler School of Physics and Astronomy, Tel Aviv University, Tel Aviv, Israel; 201 Department of Physics, Aristotle University of Thessaloniki, Thessaloniki, Greece; 202 International Center for Elementary Particle Physics and Department of Physics, The University of Tokyo, Tokyo, Japan; 203 Graduate School of Science and Technology, Tokyo Metropolitan University, Tokyo, Japan; 204 Department of Physics, Tokyo Institute of Technology, Tokyo, Japan; 205 Department of Physics, University of Toronto, Toronto, ON Canada; 206 TRIUMF, Vancouver, BC Canada; 207 Department of Physics and Astronomy, York University, Toronto, ON Canada; 208 Faculty of Pure and Applied Sciences, University of Tsukuba, Tsukuba, Japan; 209 Department of Physics and Astronomy, Tufts University, Medford, MA USA; 210 Centro de Investigaciones, Universidad Antonio Narino, Bogota, Colombia; 211 Department of Physics and Astronomy, University of California Irvine, Irvine, CA USA; 212 INFN Gruppo Collegato di Udine, Sezione di Trieste, Udine, Italy; 213 ICTP, Trieste, Italy; 214 Dipartimento di Chimica, Fisica e Ambiente, Università di Udine, Udine, Italy; 215 Department of Physics, University of Illinois, Urbana, IL USA; 216 Department of Physics and Astronomy, University of Uppsala, Uppsala, Sweden; 217 Instituto de Física Corpuscular (IFIC) and Departamento de Física Atómica, Molecular y Nuclear and Departamento de Ingeniería Electrónica and Instituto de Microelectrónica de Barcelona (IMB-CNM), University of Valencia and CSIC, Valencia, Spain; 218 Department of Physics, University of British Columbia, Vancouver, BC Canada; 219 Department of Physics and Astronomy, University of Victoria, Victoria, BC Canada; 220 Department of Physics, University of Warwick, Coventry, UK; 221 Waseda University, Tokyo, Japan; 222 Department of Particle Physics, The Weizmann Institute of Science, Rehovot, Israel; 223 Department of Physics, University of Wisconsin, Madison, WI USA; 224 Fakultät für Physik und Astronomie, Julius-Maximilians-Universität, Würzburg, Germany; 225 Fachbereich C Physik, Bergische Universität Wuppertal, Wuppertal, Germany; 226 Department of Physics, Yale University, New Haven, CT USA; 227 Yerevan Physics Institute, Yerevan, Armenia; 228 Centre de Calcul de l’Institut National de Physique Nucléaire et de Physique des Particules (IN2P3), Villeurbanne, France; 229 CERN, 1211 Geneva 23, Switzerland

## Abstract

The large rate of multiple simultaneous proton–proton interactions, or pile-up, generated by the Large Hadron Collider in Run 1 required the development of many new techniques to mitigate the adverse effects of these conditions. This paper describes the methods employed in the ATLAS experiment to correct for the impact of pile-up on jet energy and jet shapes, and for the presence of spurious additional jets, with a primary focus on the large 20.3 $\mathrm{fb}^{-1}$ data sample collected at a centre-of-mass energy of $\sqrt{s} = 8~\mathrm {TeV}$. The energy correction techniques that incorporate sophisticated estimates of the average pile-up energy density and tracking information are presented. Jet-to-vertex association techniques are discussed and projections of performance for the future are considered. Lastly, the extension of these techniques to mitigate the effect of pile-up on jet shapes using subtraction and grooming procedures is presented.

## Introduction

The success of the proton–proton ($pp$) operation of the Large Hadron Collider (LHC) at $\sqrt{s} = 8~\mathrm {TeV}$ led to instantaneous luminosities of up to $7.7\times 10^{33}$ cm$^{-2}$ s$^{-1}$ at the beginning of a fill. Consequently, multiple $pp$ interactions occur within each bunch crossing. Averaged over the full data sample, the mean number of such simultaneous interactions (pile-up) is approximately 21. These additional collisions are uncorrelated with the hard-scattering process that typically triggers the event and can be approximated as contributing a background of soft energy depositions that have particularly adverse and complex effects on jet reconstruction. Hadronic jets are observed as groups of topologically related energy deposits in the ATLAS calorimeters, and therefore pile-up affects the measured jet energy and jet structure observables. Pile-up interactions can also directly generate additional jets. The production of such *pile-up jets* can occur from additional $2\rightarrow 2$ interactions that are independent of the hard-scattering and from contributions due to soft energy deposits that would not otherwise exceed the threshold to be considered a jet. An understanding of all of these effects is therefore critical for precision measurements as well as searches for new physics.

**Fig. 1:**
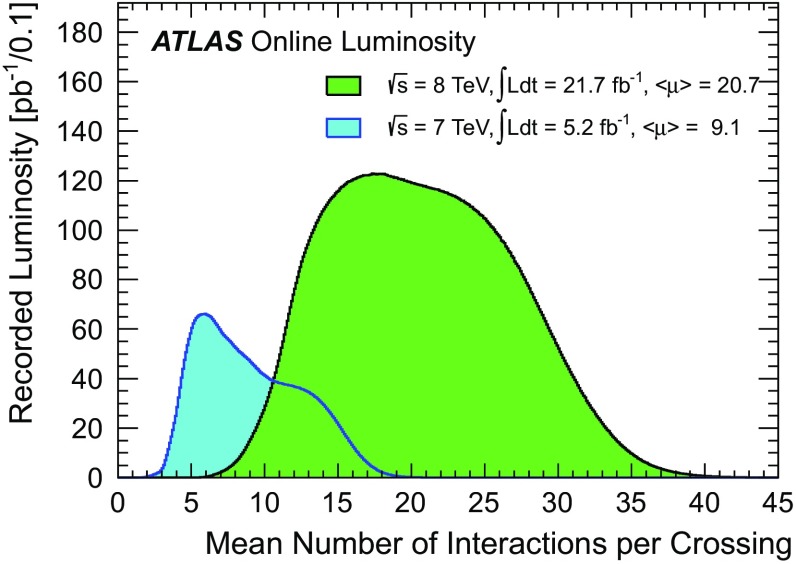
The luminosity-weighted distribution of the mean number of interactions per bunch crossing for the 2011 ($\sqrt{s} = 7~\mathrm {TeV}$) and 2012 ($\sqrt{s} = 8~\mathrm {TeV}$) $pp$ data samples

The expected amount of pile-up ($\mu$) in each bunch crossing is related to the instantaneous luminosity ($\mathcal L _0$) by the following relationship:

1 $$\begin{aligned} \mu = \frac{\mathcal L _0 \sigma _{\mathrm {inelastic}} }{n_\mathrm{c} \, f_\mathrm {rev}} \end{aligned}$$

where $n_\mathrm{c}$ is the number of colliding bunch pairs in the LHC, $f_\mathrm {rev} = 11.245$ kHz is the revolution frequency [1], and $\sigma _{\mathrm {inelastic}}$ is the $pp$ inelastic cross section. When the instantaneous luminosity is measured by integrating over many bunch crossings, Eq. (1) yields the average number of interactions per crossing, or $\langle \mu \rangle$. The so-called *in-time pile-up* due to additional $pp$ collisions within a single bunch crossing can also be accompanied by *out-of-time pile-up* due to signals from collisions in other bunch crossings. This occurs when the detector and/or electronics integration time is significantly larger than the time between crossings, as is the case for the liquid-argon (LAr) calorimeters in the ATLAS detector. The measured detector response as a function of $\langle \mu \rangle$ in such cases is sensitive to the level of out-of-time pile-up. The distributions of $\langle \mu \rangle$ for both the $\sqrt{s} = 7~\mathrm {TeV}$ and $\sqrt{s} = 8~\mathrm {TeV}$ runs (collectively referred to as Run 1) are shown in Fig. 1. The spacing between successive proton bunches was 50 ns for the majority of data collected during Run 1. This bunch spacing is decreased to 25 ns for LHC Run 2. Out-of-time pile-up contributions are likely to increase with this change. However, the LAr calorimeter readout electronics are also designed to provide an optimal detector response for a 25 ns bunch spacing scenario, and thus the relative impact of the change to 25 ns may be mitigated, particularly in the case of the calorimeter response (see Sect. 2).

The different responses of the individual ATLAS subdetector systems to pile-up influence the methods used to mitigate its effects. The sensitivity of the calorimeter energy measurements to multiple bunch crossings, and the LAr EM calorimeter in particular, necessitates correction techniques that incorporate estimates of the impact of both in-time and out-of-time pile-up. These techniques use the average deposited energy density due to pile-up as well as track-based quantities from the inner tracking detector (ID) such as the number of reconstructed primary vertices ($N_{\mathrm{PV}}$) in an event. Due to the fast response of the silicon tracking detectors, this quantity is not affected by out-of-time pile-up, to a very good approximation.

Resolving individual vertices using the ATLAS ID is a critical task in accurately determining the origin of charged-particle tracks that point to energy deposits in the calorimeter. By identifying tracks that originate in the hard-scatter primary vertex, jets that contain significant contamination from pile-up interactions can be rejected. These approaches provide tools for reducing or even obviating the effects of pile-up on the measurements from individual subdetector systems used in various stages of the jet reconstruction. The result is a robust, stable jet definition, even at very high luminosities.

The first part of this paper describes the implementation of methods to partially suppress the impact of signals from pile-up interactions on jet reconstruction and to directly estimate event-by-event pile-up activity and jet-by-jet pile-up sensitivity, originally proposed in Ref. [2]. These estimates allow for a sophisticated pile-up subtraction technique in which the four-momentum of the jet and the jet shape are corrected event-by-event for fluctuations due to pile-up, and whereby jet-by-jet variations in pile-up sensitivity are automatically accommodated. The performance of these new pile-up correction methods is assessed and compared to previous pile-up corrections based on the number of reconstructed primary vertices and the instantaneous luminosity [3, 4]. Since the pile-up subtraction is the first step of the jet energy scale (JES) correction in ATLAS, these techniques play a crucial role in establishing the overall systematic uncertainty of the jet energy scale. Nearly all ATLAS measurements and searches for physics beyond the Standard Model published since the end of the 2012 data-taking period utilise these methods, including the majority of the final Run 1 Higgs cross section and coupling measurements [5–9].

The second part of this paper describes the use of tracks to assign jets to the hard-scatter interaction. By matching tracks to jets, one obtains a measure of the fraction of the jet energy associated with a particular primary vertex. Several track-based methods allow the rejection of spurious calorimeter jets resulting from local fluctuations in pile-up activity, as well as real jets originating from single pile-up interactions, resulting in improved stability of the reconstructed jet multiplicity against pile-up. Track-based methods to reject pile-up jets are applied after the full chain of JES corrections, as pile-up jet tagging algorithms.

The discussion of these approaches proceeds as follows. The ATLAS detector is described in Sect. 2 and the data and Monte Carlo simulation samples are described in Sect. 3. Section 4 describes how the inputs to jet reconstruction are optimised to reduce the effects of pile-up on jet constituents. Methods for subtracting pile-up from jets, primarily focusing on the impacts on calorimeter-based measurements of jet kinematics and jet shapes, are discussed in Sect. 5. Approaches to suppressing the effects of pile-up using both the subtraction techniques and charged-particle tracking information are then presented in Sect. 6. Lastly, techniques that aim to correct jets by actively removing specific energy deposits that are due to pile-up, are discussed in Sect. 7.

## The ATLAS detector

The ATLAS detector [10, 11] provides nearly full solid angle coverage around the collision point with an inner tracking system covering the pseudorapidity range $|\eta |<2.5$,^1^ electromagnetic and hadronic calorimeters covering $|\eta |<4.9$, and a muon spectrometer covering $|\eta |<2.7$.

The ID comprises a silicon pixel tracker closest to the beamline, a microstrip silicon tracker, and a straw-tube transition radiation tracker at radii up to 108 cm. These detectors are layered radially around each other in the central region. A thin superconducting solenoid surrounding the tracker provides an axial 2 T field enabling the measurement of charged-particle momenta. The overall ID acceptance spans the full azimuthal range in $\phi$ for particles originating near the nominal LHC interaction region [12–14]. Due to the fast readout design of the silicon pixel and microstrip trackers, the track reconstruction is only affected by in-time pile-up. The efficiency to reconstruct charged hadrons ranges from 78 % at $p_{\text {T}} ^{\mathrm {track}}=500$ MeV to more than 85 % above $10~\mathrm {GeV}$, with a transverse impact parameter ($d_0$) resolution of 10 $\upmu$m for high-momentum particles in the central region. For jets with $p_{\text {T}}$ above approximately $500~\mathrm {GeV}$, the reconstruction efficiency for tracks in the core of the jet starts to degrade because these tracks share many clusters in the pixel tracker, creating ambiguities when matching the clusters with track candidates, and leading to lost tracks.

The high-granularity EM and hadronic calorimeters are composed of multiple subdetectors spanning $|\eta |\le 4.9$. The EM barrel calorimeter uses a LAr active medium and lead absorbers. In the region $|\eta | < 1.7$, the hadronic (Tile) calorimeter is constructed from steel absorber and scintillator tiles and is separated into barrel ($|\eta |<1.0$) and extended barrel ($0.8<|\eta |<1.7$) sections. The calorimeter end-cap ($1.375<|\eta |<3.2$) and forward ($3.1<|\eta |<4.9$) regions are instrumented with LAr calorimeters for EM and hadronic energy measurements. The response of the calorimeters to single charged hadrons—defined as the energy (*E*) reconstructed for a given charged hadron momentum (*p*), or *E* / *p*—ranges from 20 to 80 % in the range of charged hadron momentum between 1–30 GeV and is well described by Monte Carlo (MC) simulation [15]. In contrast to the pixel and microstrip tracking detectors, the LAr calorimeter readout is sensitive to signals from the preceding 12 bunch crossings during 50 ns bunch spacing operation [16, 17]. For the 25 ns bunch spacing scenario expected during Run 2 of the LHC, this increases to 24 bunch crossings. The LAr calorimeter uses bipolar shaping with positive and negative output which ensures that the average signal induced by pile-up averages to zero in the nominal 25 ns bunch spacing operation. Consequently, although the LAr detector will be exposed to more out-of-time pile-up in Run 2, the signal shaping of the front-end electronics is optimised for this shorter spacing [16, 18], and is expected to cope well with the change. The fast readout of the Tile calorimeter, however, makes it relatively insensitive to out-of-time pile-up  [19]. The LAr barrel has three EM layers longitudinal in shower depth (EM1, EM2, EM3), whereas the LAr end-cap has three EM layers (EMEC1, EMEC2, EMEC3) in the range $1.5<|\eta |<2.5$, two layers in the range $2.5<|\eta |<3.2$ and four hadronic layers (HEC1, HEC2, HEC3, HEC4). In addition, there is a pre-sampler layer in front of the LAr barrel and end-cap EM calorimeter (PS). The transverse segmentation of both the EM and hadronic LAr end-caps is reduced in the region between $2.5<|\eta |<3.2$ compared to the barrel layers. The forward LAr calorimeter has one EM layer (FCal1) and two hadronic layers (FCal2, FCal3) with transverse segmentation similar to the more forward HEC region. The Tile calorimeter has three layers longitudinal in shower depth (Tile1, Tile2, Tile3) as well as scintillators in the gap region spanning ($0.85<|\eta |<1.51$) between the barrel and extended barrel sections.

## Data and Monte Carlo samples

This section provides a description of the data selection and definitions of objects used in the analysis (Sect. 3.1) as well as of the simulated event samples to which the data are compared (Sect. 3.2).

### Object definitions and event selection

The full 2012 $pp$ data-taking period at a centre-of-mass energy of $\sqrt{s} = 8~\mathrm {TeV}$ is used for these measurements presented here. Events are required to meet baseline quality criteria during stable LHC running periods. The ATLAS data quality (DQ) criteria reject data with significant contamination from detector noise or issues in the read-out [20] based upon individual assessments for each subdetector. These criteria are established separately for the barrel, end-cap and forward regions, and they differ depending on the trigger conditions and reconstruction of each type of physics object (for example jets, electrons and muons). The resulting dataset corresponds to an integrated luminosity of $20.3 \pm 0.6$ $\mathrm{fb}^{-1}$ following the methodology described in Ref. [21].

To reject non-collision backgrounds [22], events are required to contain at least one primary vertex consistent with the LHC beam spot, reconstructed from at least two tracks each with $p_{\text {T}} ^{\mathrm {track}}>400$ MeV. The primary hard-scatter vertex is defined as the vertex with the highest $\sum (p_{\text {T}} ^{\mathrm {track}})^2$. To reject rare events contaminated by spurious signals in the detector, all anti-$k_{t}$  [23, 24] jets with radius parameter $R=0.4$ and $p_{\text {T}} ^\mathrm {jet} >20 \mathrm {GeV}$ (see below) are required to satisfy the jet quality requirements that are discussed in detail in Ref. [22] (and therein referred to as the “looser” selection). These criteria are designed to reject non-collision backgrounds and significant transient noise in the calorimeters while maintaining an efficiency for good-quality events greater than 99.8 % with as high a rejection of contaminated events as possible. In particular, this selection is very efficient in rejecting events that contain fake jets due to calorimeter noise.

Hadronic jets are reconstructed from calibrated three-dimensional topo-clusters [25]. Clusters are constructed from calorimeter cells that are grouped together using a topological clustering algorithm. These objects provide a three-dimensional representation of energy depositions in the calorimeter and implement a nearest-neighbour noise suppression algorithm. The resulting topo-clusters are classified as either electromagnetic or hadronic based on their shape, depth and energy density. Energy corrections are then applied to the clusters in order to calibrate them to the appropriate energy scale for their classification. These corrections are collectively referred to as *local cluster weighting*, or LCW, and jets that are calibrated using this procedure are referred to as LCW jets [4].

Jets can also be built from charged-particle tracks (track-jets) using the identical anti-$k_{t}$ algorithm as for jets built from calorimeter clusters. Tracks used to construct track-jets have to satisfy minimal quality criteria, and they are required to be associated with the hard-scatter vertex.

The jets used for the analyses presented here are primarily found and reconstructed using the anti-$k_{t}$ algorithm with radius parameters $R = 0.4, 0.6$ and 1.0. In some cases, studies of *groomed* jets are also performed, for which algorithms are used to selectively remove constituents from a jet. Groomed jets are often used in searches involving highly Lorentz-boosted massive objects such as *W* / *Z* bosons [26] or top quarks [27]. Unless noted otherwise, the jet trimming algorithm [28] is used for groomed jet studies in this paper. The procedure implements a $k_{t}$ algorithm [29, 30] to create small *subjets* with a radius $R_\mathrm{sub} =0.3$. The ratio of the $p_{\text {T}}$ of these subjets to that of the jet is used to remove constituents from the jet. Any subjets with $p_{\mathrm{T}i}/p_{\text {T}} ^\mathrm {jet} < f_\mathrm{cut}$ are removed, where $p_{\mathrm{T}i}$ is the transverse momentum of the *i*th subjet, and $f_\mathrm{cut} =0.05$ is determined to be an optimal setting for improving mass resolution, mitigating the effects of pile-up, and retaining substructure information [31]. The remaining constituents form the trimmed jet.

The energy of the reconstructed jet may be further corrected using subtraction techniques and multiplicative jet energy scale correction factors that are derived from MC simulation and validated with the data [3, 4]. As discussed extensively in Sect. 5, subtraction procedures are critical to mitigating the jet energy scale dependence on pile-up. Specific jet energy scale correction factors are then applied after the subtraction is performed. The same corrections are applied to calorimeter jets in MC simulation and data to ensure consistency when direct comparisons are made between them.

Comparisons are also made to jets built from particles in the MC generator’s event record (“truth particles”). In such cases, the inputs to jet reconstruction are stable particles with a lifetime of at least 10 ps (excluding muons and neutrinos). Such jets are referred to as *generator-level jets* or *truth-particle jets* and are to be distinguished from *parton-level jets*. Truth-particle jets represent the measurement for a hermetic detector with perfect resolution and scale, without pile-up, but including the underlying event.

Trigger decisions in ATLAS are made in three stages: Level-1, Level-2 and the Event Filter. The Level-1 trigger is implemented in hardware and uses a subset of detector information to reduce the event rate to a design value of at most 75 kHz. This is followed by two software-based triggers, Level-2 and the Event Filter, which together reduce the event rate to a few hundred Hz. The measurements presented in this paper primarily use single-jet triggers. The rate of events in which the highest transverse momentum jet is less than about $400~\mathrm {GeV}$ is too high to record more than a small fraction of them. The triggers for such events are therefore pre-scaled to reduce the rates to an acceptable level in an unbiased manner. Where necessary, analyses compensate for the pre-scales by using weighted events based upon the pre-scale setting that was active at the time of the collision.

### Monte Carlo simulation

Two primary MC event generator programs are used for comparison to the data. PYTHIA 8.160 [32] with the ATLAS A2 tunable parameter set (tune) [33] and the CT10 NLO parton distribution function (PDF) set [34] is used for the majority of comparisons. Comparisons are also made to the HERWIG++ 2.5.2 [35] program using the CTEQ6L1 [36] PDF set along with the UE7-2 tune [37], which is tuned to reproduce underlying-event data from the LHC experiments. MC events are passed through the full GEANT4  [38] detector simulation of ATLAS [39] after the simulation of the parton shower and hadronisation processes. Identical reconstruction and trigger, event, quality, jet and track selection criteria are then applied to both the MC simulation and to the data.

In some cases, additional processes are used for comparison to data. The $Z$ boson samples used for the validation studies are produced with the POWHEG-BOX v1.0 generator [40–42] and the SHERPA 1.4.0 [43] generator, both of which provide NLO matrix elements for inclusive $Z$ boson production. The CT10 NLO PDF set is also used in the matrix-element calculation for these samples. The modelling of the parton shower, multi-parton interactions and hadronisation for events generated using POWHEG-BOX is provided by PYTHIA 8.163 with the AU2 underlying-event tune [33] and the CT10 NLO PDF set. These MC samples are thus referred to as POWHEG+PYTHIA  8 samples. PYTHIA is in turn interfaced with PHOTOS [44] for the modelling of QED final-state radiation.

Pile-up is simulated for all samples by overlaying additional soft $pp$ collisions which are also generated with PYTHIA 8.160 using the ATLAS A2 tune and the MSTW2008LO PDF set [45]. These additional events are overlaid onto the hard-scattering events according to the measured distribution of the average number $\langle \mu \rangle$ of $pp$ interactions per bunch crossing from the luminosity detectors in ATLAS [21, 46] using the full 8 TeV data sample, as shown in Fig. 1. The proton bunches were organised in four trains of 36 bunches with a 50 ns spacing between the bunches. Therefore, the simulation also contains effects from out-of-time pile-up. The effect of this pile-up history for a given detector system is then determined by the size of the readout time window for the relevant electronics. As an example, for the central LAr calorimeter barrel region, which is sensitive to signals from the preceding 12 bunch crossings during 50 ns bunch spacing operation, the digitization window is set to 751 ns before and 101 ns after the simulated hard-scattering interaction.

## Topological clustering and cluster-level pile-up suppression

The first step for pile-up mitigation in ATLAS is at the level of the constituents used to reconstruct jets. The topological clustering algorithm incorporates a built-in pile-up suppression scheme to limit the formation of clusters produced by pile-up depositions as well as to limit the growth of clusters around highly energetic cells from hard-scatter signals. The key concept that allows this suppression is the treatment of pile-up as noise, and the use of cell energy thresholds based on their energy significance relative to the total noise.

Topological clusters are built using a three-dimensional nearest-neighbour algorithm that clusters calorimeter cells with energy significance $|E_\mathrm{cell}|/\sigma ^\mathrm{noise}>4$ for the seed, iterates among all neighbouring cells with $|E_\mathrm{cell}|/\sigma ^\mathrm{noise}>2$, and that finally adds one additional layer of cells $|E_\mathrm{cell}|/\sigma ^\mathrm{noise}>0$ when no further nearest-neighbours exceed the $2\sigma$ threshold at the boundary (not allowed to extend to next-to-nearest neighbours). The total cell noise, $\sigma ^\mathrm{noise}$, is the sum in quadrature of the cell noise due to the readout electronics and the cell noise that is due to pile-up ($\sigma _\mathrm{pile-up}^\mathrm{noise}$). The pile-up noise for a given cell is evaluated from Monte Carlo simulation and is defined to be the RMS of the energy distribution resulting from pile-up particles for a given number of $pp$ collisions per bunch crossing (determined by $\langle \mu \rangle$) and a given bunch spacing $\Delta t$. It is technically possible to adjust the pile-up noise for specific data-taking periods depending on $\langle \mu \rangle$, but it was kept fixed for the entire Run 1 $8 \mathrm {TeV}$ dataset.

By adjusting the pile-up noise value, topological clustering partially suppresses the formation of clusters created by pile-up fluctuations, and it reduces the number of cells included in jets. Raising the pile-up noise value effectively increases the threshold for cluster formation and growth, significantly reducing the effects of pile-up on the input signals to jet reconstruction.

**Fig. 2:**
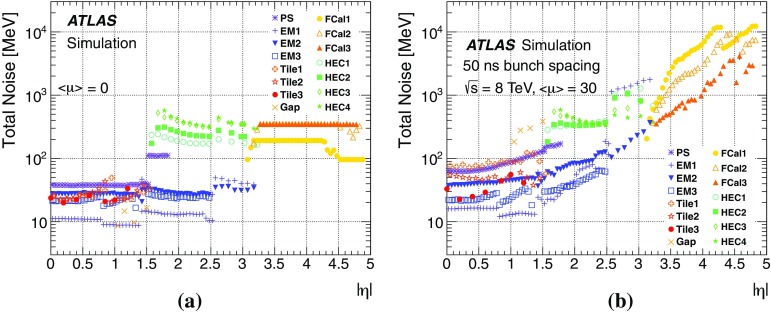
**a** Per-cell electronic noise ($\langle \mu \rangle =0$) and **b** total noise per cell at high luminosity corresponding to $\langle \mu \rangle =30$ interactions per bunch crossing with a bunch spacing of $\Delta t = 50$ ns, in MeV, for each calorimeter layer. The *different colours* indicate the noise in the pre-sampler (PS), the up to three layers of the LAr calorimeter (EM), the up to three layers of the Tile calorimeter (Tile), the four layers of the hadronic end-cap calorimeter (HEC), and the three layers of the forward calorimeter (FCal). The total noise, $\sigma ^\mathrm{noise}$, is the sum in quadrature of electronic noise and the expected RMS of the energy distribution corresponding to a single cell

Figure 2 shows the electronic and pile-up noise contributions to cells that are used to define the thresholds for the topological clustering algorithm. In events with an average of 30 additional pile-up interactions ($\langle \mu \rangle =30$), the noise from pile-up depositions is approximately a factor of 2 larger than the electronic noise for cells in the central electromagnetic calorimeter, and it reaches $10~\mathrm {GeV}$ in FCal1 and FCal2. This high threshold in the forward region translates into a reduced topo-cluster occupancy due to the coarser segmentation of the forward calorimeter, and thus a smaller probability that a given event has a fluctuation beyond $4\sigma$. The implications of this behaviour for the pile-up $p_{\text {T}}$ density estimation are discussed in Sect. 5.1.

The value of $\langle \mu \rangle$ at which $\sigma _\mathrm{pile-up}^\mathrm{noise}$ is evaluated for a given data-taking period is chosen to be high enough that the number of clusters does not grow too large due to pile-up and at the same time low enough to retain as much signal as possible. For a Gaussian noise distribution the actual $4\sigma$ seed threshold leads to an increase in the number of clusters by a factor of 5 if the noise is underestimated by 10 %. Therefore $\sigma _\mathrm{pile-up}^\mathrm{noise}$ was set to the pile-up noise corresponding to the largest expected $\langle \mu \rangle$ rather than the average or the lowest expected value. For 2012 (2011) pile-up conditions, $\sigma _\mathrm{pile-up}^\mathrm{noise}$ was set to the value of $\sigma _\mathrm{pile-up}^\mathrm{noise}$ corresponding to $\langle \mu \rangle =30$ ($\langle \mu \rangle =8$).

The local hadron calibration procedure for clusters depends on the value of $\sigma ^\mathrm{noise}$ since this choice influences the cluster size and thus the shape variables used in the calibration. Therefore, the calibration constants are re-computed for each $\sigma ^\mathrm{noise}$ configuration. For this reason, a single, fixed value of $\sigma ^\mathrm{noise}$ is used for entire data set periods in order to maintain consistent conditions.

## Pile-up subtraction techniques and results

The independence of the hard-scattering process from additional pile-up interactions in a given event results in positive or negative shifts to the reconstructed jet kinematics and to the jet shape. This motivates the use of subtraction procedures to remove these contributions to the jet. Early subtraction methods [3, 4] for mitigating the effects of pile-up on the jet transverse momentum in ATLAS relied on an average offset correction ($\langle \mathcal {O} ^\mathrm{jet} \rangle$),

2 $$\begin{aligned} p_{\text {T}} ^\mathrm {corr} = p_{\text {T}} ^\mathrm {jet}- \langle \mathcal {O} ^\mathrm{jet} (\langle \mu \rangle ,N_{\mathrm{PV}},\eta )\rangle . \end{aligned}$$

In these early approaches, $\langle \mathcal {O} ^\mathrm{jet} \rangle$ is determined from in-situ studies or MC simulation and represents an average offset applied to the jet $p_{\text {T}}$. This offset is parametrised as a function of $\eta$, $N_{\mathrm{PV}}$ and $\langle \mu \rangle$. Such methods do not fully capture the fluctuations of the pile-up energy added to the calorimeter on an event-by-event basis; that component is only indirectly estimated from its implicit dependence on $N_{\mathrm{PV}}$. Moreover, no individual jet’s information enters into this correction and thus jet-by-jet fluctuations in the actual offset of that particular jet $p_{\text {T}}$, $\mathcal {O} ^\mathrm{jet}$, or the jet shape, cannot be taken into account. Similar methods have also been pursued by the CMS collaboration [47], as well a much more complex approaches that attempt to mitigate the effects of pile-up prior to jet reconstruction [48, 49].

The approach adopted for the final Run 1 ATLAS jet energy scale [4] is to estimate $\mathcal {O} ^\mathrm{jet}$ on an event-by-event basis. To accomplish this, a measure of the jet’s susceptibility to soft energy depositions is needed in conjunction with a method to estimate the magnitude of the effect on a jet-by-jet and event-by-event basis. A natural approach is to define a jet *area* ($A^{\mathrm {jet}}$) [50] in $\eta$–$\phi$ space along with a pile-up $p_{\text {T}}$ density, $\rho$. The offset can then be determined dynamically for each jet [2] using

3 $$\begin{aligned} \mathcal {O} ^\mathrm{jet} = \rho \times A^{\mathrm {jet}}. \end{aligned}$$

Nearly all results published by ATLAS since 2012 have adopted this technique for correcting the jet kinematics for pile-up effects. The performance of this approach, as applied to both the jet kinematics and the jet shape, is discussed below.

### Pile-up event $p_{\text {T}}$ density $\rho$

One of the key parameters in the pile-up subtraction methods presented in this paper is the estimated pile-up $p_{\text {T}}$ density characterised by the observable $\rho$. The pile-up $p_{\text {T}}$ density of an event can be estimated as the median of the distribution of the density of many $k_{t}$ jets, constructed with no minimum $p_{\text {T}}$ threshold [29, 30] in the event. Explicitly, this is defined as

4 $$\begin{aligned} \rho = \mathrm {median}\left\{ \frac{p_{\mathrm {T}, i}^\mathrm {jet}}{A^{\mathrm {jet}} _{i}}\right\} , \end{aligned}$$

where each $$k_{t}$$ jet *i* has transverse momentum $$p_{\mathrm {T}, i}^\mathrm {jet}$$ and area $$A^{\mathrm {jet}} _{i}$$, and it is defined with a nominal radius parameter $$R_{k_{t}} =0.4$$. The chosen radius parameter value is the result of a dedicated optimisation study, balancing two competing effects: the sensitivity to biases from hard-jet contamination in the $$\rho $$ calculation when $$R_{k_{t}} $$ is large, and statistical fluctuations when $$R_{k_{t}} $$ is small. The sensitivity to the chosen radius value is not large, but measurably worse performance was observed for radius parameters larger than 0.5 and smaller than 0.3.

**Fig. 3:**
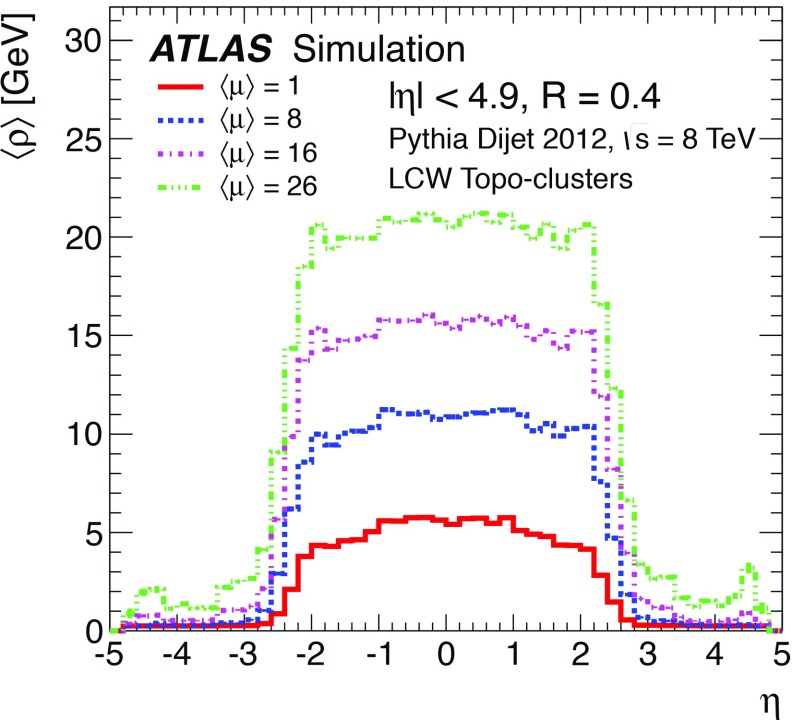
The mean estimated pile-up $p_{\text {T}}$ density, $\rho$ as a function of $\eta$, in simulated PYTHIA 8 dijet events

The use of the $k_{t}$ algorithm in Eq. (4) is motivated by its sensitivity to soft radiation and thus no minimum $p_{\text {T}}$ selection is applied to the $k_{t}$ jets that are used. In ATLAS, the inputs to the $k_{t}$ jets used in the $\rho$ calculation are positive-energy calorimeter topo-clusters within $|\eta | \le 2.0$. The $\eta$ range chosen for calculating $\rho$ is motivated by the calorimeter occupancy, which is low in the forward region relative to the central region. The cause of the low occupancy in the forward region is complex and is intrinsically related to the calorimeter segmentation and response. The coarser calorimeter cell size at higher $|\eta |$ [10], coupled with the noise suppression inherent in topological clustering, plays a large role. Since topo-clusters are seeded according to significance relative to (electronic and pile-up) noise rather than an absolute threshold, having a larger number of cells (finer segmentation) increases the probability that the energy of one cell fluctuates up to a significant value due to (electronic or pile-up) noise. With the coarser segmentation in the end-cap and forward regions beginning near $|\eta |= 2.5$ (see Fig. 2), this probability becomes smaller, and clusters are predominantly seeded only by the hard-scatter signal. In addition, the likelihood that hadronic showers overlap in a single cell increases along with the probability that fluctuations in the calorimeter response cancel, which affects the energy deposited in the cell. The mean $\rho$ measured as a function of $\eta$ is shown in Fig. 3. The measurements are made in narrow strips in $\eta$ which are $\Delta \eta = 0.7$ wide and shifted in steps of $\delta \eta = 0.1$ from $\eta = - 4.9$ to 4.9. The $\eta$ reported in Fig. 3 is the central value of each strip. The measured $\rho$ in each strip quickly drops to nearly zero beyond $|\eta |\simeq 2$. Due to this effectively stricter suppression in the forward region, a calculation of $\rho$ in the central region gives a more meaningful measure of the pile-up activity than the median over the entire $\eta$ range, or an $\eta$-dependent $\rho$ calculated in slices across the calorimeter.

**Fig. 4:**
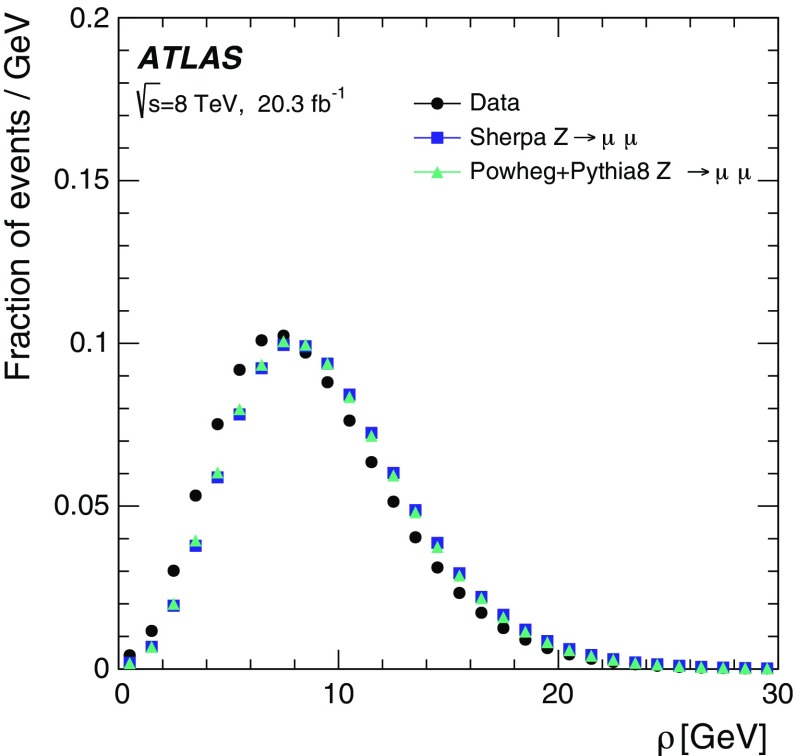
The distribution of estimated pile-up $p_{\text {T}}$ density, $\rho$, in $Z (\rightarrow \mu \mu )$+jets events using data and two independent MC simulation samples (SHERPA and POWHEG+PYTHIA  8). Both MC generators use the same pile-up simulation model (PYTHIA  8.160), and this model uses the $\langle \mu \rangle$ distribution for $8 \mathrm {TeV}$ data shown in Fig. 1. $\rho$ is calculated in the central region using topo-clusters with positive energy within $|\eta | \le 2.0$

Distributions of $\rho$ in both data and MC simulation are presented in Fig. 4 for SHERPA and POWHEG+PYTHIA  8. Both MC generators use the same pile-up simulation model. The event selection used for these distributions corresponds to $Z (\rightarrow \mu \mu )$+jets events where a $Z$ boson ($p_{\text {T}} ^{Z} >30$ GeV) and a jet ($|\eta |<2.5$ and $p_{\text {T}} >20$ GeV) are produced back-to-back ($\Delta \phi (Z,\mathrm{leading~jet})>2.9$). Both MC simulations slightly overestimate $\rho$, but agree well with each other. Small differences between the MC simulations can be caused by different modelling of the soft jet spectrum associated with the hard-scattering and the underlying event.

Since $\rho$ is computed event-by-event, separately for data and MC, a key advantage of the jet area subtraction is that it reduces the pile-up uncertainty from detector mismodelling effects. This is because different values of $\rho$ are determined in data and simulation depending on the measured pile-up activity rather than using a predicted value for $\rho$ based on MC simulations.

### Pile-up energy subtraction

The median $p_{\text {T}}$ density $\rho$ provides a direct estimate of the global pile-up activity in each event, whereas the jet area provides an estimate of an individual jet’s susceptibility to pile-up. Equation (2) can thus be expressed on a jet-by-jet basis using Eq. (3) instead of requiring an average calculation of the offset, $\langle \mathcal {O} \rangle$. This yields the following pile-up subtraction scheme:

5 $$\begin{aligned} p_{\text {T}} ^\mathrm {corr} = p_{\text {T}} ^\mathrm {jet}- \rho \times A^{\mathrm {jet}}. \end{aligned}$$

There are two ways in which pile-up can contribute energy to an event: either by forming new clusters, or by overlapping with signals from the hard-scattering event. Because of the noise suppression inherent in topological clustering, only pile-up signals above a certain threshold can form separate clusters. Low-energy pile-up deposits can thus only contribute measurable energy to the event if they overlap with other deposits that survive noise suppression. The probability of overlap is dependent on the transverse size of EM and hadronic showers in the calorimeter, relative to the size of the calorimeter cells. Due to fine segmentation, pile-up mainly contributes extra clusters in the central regions of the calorimeter where $\rho$ is calculated ($|\eta |\lesssim 2$).

As discussed in Sect. 2, the details of the readout electronics for the LAr calorimeter can result in signals associated with out-of-time pile-up activity. If out-of-time signals from earlier bunch crossings are isolated from in-time signals, they may form negative energy clusters, which are excluded from jet reconstruction and the calculation of $\rho$. However, overlap between the positive jet signals and out-of-time activity results in both positive and negative modulation of the jet energy. Due to the long negative component of the LAr pulse shape, the probability is higher for an earlier bunch crossing to negatively contribute to signals from the triggered event than a later bunch crossing to contribute positively. This feature results in a negative dependence of the jet $p_{\text {T}}$ on out-of-time pile-up. Such overlap is more probable at higher $|\eta |$, due to coarser segmentation relative to the transverse shower size. In addition, the length of the bipolar pulse is shorter in the forward calorimeters, which results in larger fluctuations in the out-of-time energy contributions to jets in the triggered event since the area of the pulse shape must remain constant. As a result, forward jets have enhanced sensitivity to out-of-time pile-up due to the larger impact of fluctuations of pile-up energy depositions in immediately neighbouring bunch crossings.

Since the $\rho$ calculation is dominated by lower-occupancy regions in the calorimeter, the sensitivity of $\rho$ to pile-up does not fully describe the pile-up sensitivity of the high-occupancy region at the core of a high-$p_{\text {T}}$ jet. The noise suppression provided by the topological clustering procedure has a smaller impact in the dense core of a jet where significant nearby energy deposition causes a larger number of small signals to be included in the final clusters than would otherwise be possible. Furthermore, the effects of pile-up in the forward region are not well described by the median $p_{\text {T}}$ density as obtained from positive clusters in the central region. A residual correction is therefore necessary to obtain an average jet response that is insensitive to pile-up across the full $p_{\text {T}}$ range.

**Fig. 5:**
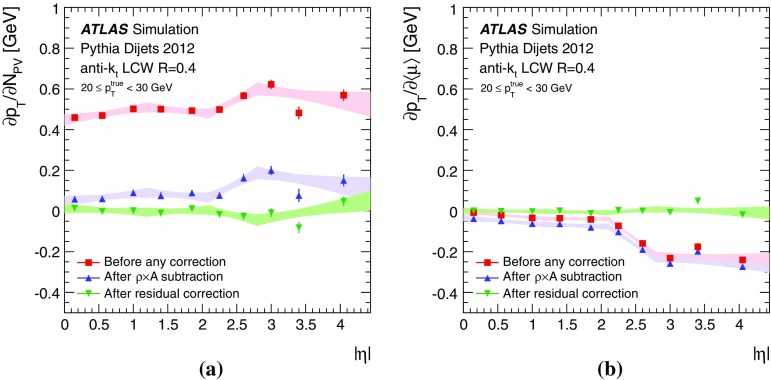
Dependence of the reconstructed jet $p_{\text {T}}$ (anti-$k_{t}$, $R = 0.4$, LCW scale) on **a** in-time pile-up measured using $N_{\mathrm{PV}}$ and **b** out-of-time pile-up measured using $\langle \mu \rangle$. In each case, the dependence of the jet $p_{\text {T}}$ is shown for three correction stages: before any correction, after the $\rho \times A^{\mathrm {jet}}$ subtraction, and after the residual correction. The error bands show the 68 % confidence intervals of the fits. The dependence was obtained by comparison with truth-particle jets in simulated dijet events, and corresponds to a truth-jet $p_{\text {T}}$ range of 20–30 GeV

Figure 5 shows the $\eta$ dependence of the transverse momentum of anti-$k_{t}$ $R = 0.4$ jets on $N_{\mathrm{PV}}$ (for fixed $\langle \mu \rangle$) and on $\langle \mu \rangle$ (for fixed $N_{\mathrm{PV}}$). Separating these dependencies probes the effects of in-time and out-of-time pile-up, respectively, as a function of $\eta$. These results were obtained from linear fits to the difference between the reconstructed and the true jet $p_{\text {T}}$ (written as $p_{\text {T}} ^{\mathrm {reco}}- p_\mathrm{T}^\mathrm{true}$) as a function of both $N_{\mathrm{PV}}$ and $\langle \mu \rangle$. The subtraction of $\rho \times A^{\mathrm {jet}}$ removes a significant fraction of the sensitivity to in-time pile-up. In particular, the dependence decreases from nearly $0.5~\mathrm {GeV}$ per additional vertex to $\lesssim 0.2~\mathrm {GeV}$ per vertex, or a factor of 3–5 reduction in pile-up sensitivity. This reduction in the dependence of the $p_{\text {T}}$ on pile-up does not necessarily translate into a reduction of the pile-up dependence of other jet observables. Moreover, some residual dependence on $N_{\mathrm{PV}}$ remains. Figure 5b shows that $\rho \times A^{\mathrm {jet}}$ subtraction has very little effect on the sensitivity to out-of-time pile-up, which is particularly significant in the forward region. The dependence on $N_{\mathrm{PV}}$ is evaluated in bins of $\langle \mu \rangle$, and vice versa. Both dependencies are evaluated in bins of $p_\mathrm{T}^\mathrm{true}$ and $\eta$ as well. The slope of the linear fit as a function of $N_{\mathrm{PV}}$ does not depend significantly on $\langle \mu \rangle$, or vice versa, within each $(p_\mathrm{T}^\mathrm{true}, \eta )$ bin. In other words, there is no statistically significant evidence for non-linearity or cross-terms in the sensitivity of the jet $p_{\text {T}}$ to in-time or out-of-time pile-up for the values of $\langle \mu \rangle$ seen in 2012 data. A measurable effect of such non-linearities may occur with the shorter bunch spacing operation, and thus increased out-of-time pile-up effects, expected during Run 2 of the LHC. Measurements and validations of this sort are therefore important for establishing the sensitivity of this correction technique to such changes in the operational characteristics of the accelerator.

After subtracting $\rho \times A^{\mathrm {jet}}$ from the jet $p_{\text {T}}$, there is an additional subtraction of a residual term proportional to the number ($N_{\mathrm{PV}}- 1$) of reconstructed pile-up vertices, as well as a residual term proportional to $\langle \mu \rangle$ (to account for out-of-time pile-up). This residual correction is derived by comparison to truth particle jets in simulated dijet events, and it is completely analogous to the average pile-up offset correction used previously in ATLAS [4]. Due to the preceding $\rho \times A^{\mathrm {jet}}$ subtraction, the residual correction is generally quite small for jets with $|\eta | < 2.1$. In the forward region, the negative dependence of jets on out-of-time pile-up results in a significantly larger residual correction. The $\langle \mu \rangle$-dependent term of the residual correction is approximately the same size as the corresponding term in the average offset correction of Eq. (2), but the $N_{\mathrm{PV}}$-dependent term is significantly smaller. This is true even in the forward region, which shows that $\rho$ is a useful estimate of in-time pile-up activity even beyond the region in which it is calculated.

Several additional jet definitions are also studied, including larger nominal jet radii and alternative jet algorithms. Prior to the jet area subtraction, a larger sensitivity to in-time pile-up is observed for larger-area jets, as expected. Following the subtraction procedure in Eq. (5) similar results are obtained even for larger-area jet definitions. These results demonstrate that $\rho \times A^{\mathrm {jet}}$ subtraction is able to effectively reduce the impact of in-time pile-up regardless of the jet definition, although a residual correction is required to completely remove the dependence on $N_{\mathrm{PV}}$ and $\langle \mu \rangle$.

**Fig. 6:**
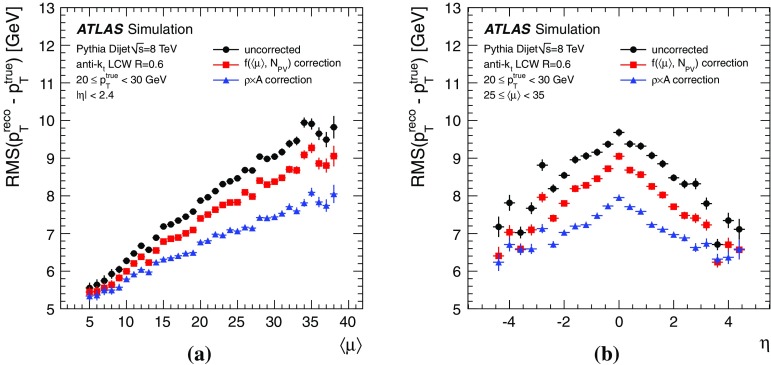
**a** RMS width of the $p_{\text {T}} ^{\mathrm {reco}}- p_\mathrm{T}^\mathrm{true}$ distribution versus $\langle \mu \rangle$ and **b** versus pseudorapidity $\eta$, for anti-$k_{t}$ $R=0.6$ jets at the LCW scale matched to truth-particle jets satisfying $20< p_\mathrm{T}^\mathrm{true} < 30 \mathrm {GeV}$, in simulated dijet events. A significant improvement is observed compared to the previous subtraction method (shown in *red*) [4]

In addition to the slope of the $p_{\text {T}}$ dependence on $N_{\mathrm{PV}}$, the RMS of the $p_{\text {T}} ^{\mathrm {reco}}- p_\mathrm{T}^\mathrm{true}$ distribution is studied as a function of $\langle \mu \rangle$ and $\eta$ in Fig. 6. For this result, anti-$k_{t}$ $R=0.6$ jets are chosen due to their greater susceptibility to pile-up and the greater challenge they therefore pose to pile-up correction algorithms. The RMS width of this distribution is an approximate measure of the jet $p_{\text {T}}$ resolution for the narrow truth-particle jet $p_{\text {T}}$ ranges used in Fig. 6. These results show that the area subtraction procedure provides an approximate 20 % reduction in the magnitude of the jet-by-jet fluctuations introduced by pile-up relative to uncorrected jets and approximately a 10 % improvement over the simple offset correction. Smaller radius $R=0.4$ jets exhibit a similar relative improvement compared to the simple offset correction. It should be noted that the pile-up activity in any given event may have significant local fluctuations similar in angular size to jets, and a global correction such as that provided by the area subtraction procedure defined in Eq. (3) cannot account for them. Variables such as the *jet vertex fraction* $\mathrm{JVF}$, *corrected* $\mathrm{JVF}$ or $\mathrm{corrJVF}$, or the *jet vertex tagger* $\mathrm{JVT}$ may be used to reject jets that result from such fluctuations in pile-up $p_{\text {T}}$ density, as described in Sect. 6.

Two methods of in-situ validation of the pile-up correction are employed to study the dependence of jet $p_{\text {T}}$ on $N_{\mathrm{PV}}$ and $\langle \mu \rangle$. The first method uses track-jets to provide a measure of the jet $p_{\text {T}}$ that is pile-up independent. This requires the presence of track-jets and so can only be used in the most central region of the detector for $|\eta | < 2.1$. It is not statistically limited. The second method exploits the $p_{\text {T}}$ balance between a reconstructed jet and a $Z$ boson, using the $p_{\text {T}} ^{Z}$ as a measure of the jet $p_{\text {T}}$. This enables an analysis over the full ($|\eta |<4.9$) range of the detector, but the extra selections applied to the jet and $Z$ boson reduce its statistical significance. The $N_{\mathrm{PV}}$ dependence must therefore be evaluated inclusively in $\langle \mu \rangle$ and vice versa. This results in a degree of correlation between the measured $N_{\mathrm{PV}}$ and $\langle \mu \rangle$ dependence.

While the pile-up residual correction is derived from simulated dijet events, the in-situ validation is done entirely using $$Z $$+jets events. In the track-jet validation, although the kinematics of the $$Z$$ boson candidate are not used directly, the dilepton system is relied upon for triggering, thus avoiding any potential bias from jet triggers.

**Fig. 7:**
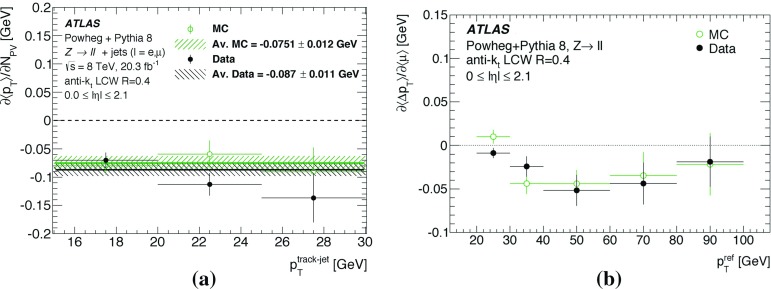
**a** $$N_{\mathrm{PV}}$$ dependence of the reconstructed $$p_{\text {T}}$$ of anti-$$k_{t}$$ $$R=0.4$$ LCW jets after the area subtraction as a function of track-jet $$p_{\text {T}}$$. **b** Validation results from $$Z $$+jets events showing the $$\langle \mu \rangle $$ dependence as a function of the $$Z$$ boson $$p_{\text {T}}$$, denoted by $$p_{\text {T}} ^{\mathrm {ref}}$$, for anti-$$k_{t}$$ $$R=0.4$$ LCW jets in the central region after the area subtraction. The* points* represent central values of the linear fit to $$\partial \langle \Delta p_{\text {T}} \rangle /\partial \langle \mu \rangle $$ and the *error bars* correspond to the associated fitting error

Figure 7a shows the results obtained when matching anti-$k_{t}$ $R=0.4$, LCW reconstructed jets to anti-$k_{t}$ $R=0.4$ track-jets. No selection is applied based on the calorimeter-based jet $p_{\text {T}}$. Good agreement is observed between data and MC simulation; however, a small overcorrection is observed in the $N_{\mathrm{PV}}$ dependence of each. For the final uncertainties on the method, this non-closure of the correction is taken as an uncertainty in the jet $p_{\text {T}}$ dependence on $N_{\mathrm{PV}}$.

In events where a $Z$ boson is produced in association with one jet, momentum conservation ensures balance between the $Z$ boson and the jet in the transverse plane. In the direct $p_{\text {T}}$ balance method, this principle is exploited by using $p_{\text {T}} ^{Z}$ as a proxy for the true jet $p_{\text {T}}$. In the case of a perfect measurement of lepton energies and provided that all particles recoiling against the $Z$ boson are included in the jet cone, the jet is expected to balance the $Z$ boson. Therefore the estimated $Z$ boson $p_{\text {T}}$ is used as the reference scale, denoted by $p_{\text {T}} ^{\mathrm {ref}}$.

Taking the mean, $\langle \Delta p_{\text {T}} \rangle$, of the ($\Delta p_{\text {T}} = p_{\text {T}}- p_{\text {T}} ^{\mathrm {ref}}$) distribution, the slope $\partial \langle \Delta p_{\text {T}} \rangle /\partial \langle \mu \rangle$ is extracted and plotted as a function of $p_{\text {T}} ^{\mathrm {ref}}$, as shown in Fig. 7b. A small residual slope is observed after the jet-area correction, which is well modelled by the MC simulation, as can be seen in Fig. 7b. The mismodelling is quantified by the maximum differences between data and MC events for both $\partial \langle \Delta p_{\text {T}} \rangle /\partial N_{\mathrm{PV}}$ and $\partial \langle \Delta p_{\text {T}} \rangle /\partial \langle \mu \rangle$. These differences (denoted by $\Delta \left( \partial \langle \Delta p_{\text {T}} \rangle /\partial N_{\mathrm{PV}} \right)$ and $\Delta \left( \partial \langle \Delta p_{\text {T}} \rangle /\partial \langle \mu \rangle \right)$) are included in the total systematic uncertainty.

The systematic uncertainties are obtained by combining the measurements from $Z$–jet balance and track-jet in-situ validation studies. In the central region ($|\eta | < 2.1$) only the track-jet measurements are used whereas $Z$–jet balance is used for $2.1< |\eta | < 4.5$. In the case of the $Z$–jet balance in the forward region, the effects of in-time and out-of-time pile-up cannot be fully decoupled. Therefore, the $N_{\mathrm{PV}}$ uncertainty is assumed to be $\eta$-independent and is thus extrapolated from the central region. In the forward region, the uncertainty on the $\langle \mu \rangle$ dependence, $\Delta \left( \partial \langle \Delta p_{\text {T}} \rangle /\partial \langle \mu \rangle \right)$, is taken to be the maximum difference between $\partial \langle \Delta p_{\text {T}} \rangle /\partial \langle \mu \rangle$ in the central region and $\partial \langle \Delta p_{\text {T}} \rangle /\partial \langle \mu \rangle$ in the forward region. In this way, the forward region $\Delta \left( \partial \langle \Delta p_{\text {T}} \rangle /\partial \langle \mu \rangle \right)$ uncertainty implicitly includes any $\eta$ dependence.

### Pile-up shape subtraction

The jet shape subtraction method [51] determines the sensitivity of jet shape observables, such as the jet width or substructure shapes, to pile-up by evaluating the sensitivity of that shape to variations in infinitesimally soft energy depositions. This variation is evaluated numerically for each jet in each event and then extrapolated to zero to derive the correction.

The procedure uses a uniform distribution of infinitesimally soft particles, or *ghosts*, that are added to the event. These ghost particles are distributed with a number density $\nu _{g}$ per unit in *y*–$\phi$ space, yielding an individual ghost area $A_{g} =1/\nu _{g}$. The four-momentum of ghost *i* is defined as

6 $$\begin{aligned} g_{\mu ,i} = g_{t} \cdot [\cos {\phi _i}, \sin {\phi _i}, \sinh {y_i}, \cosh {y_i}], \end{aligned}$$

where $$g_{t}$$ is the ghost transverse momentum (initially set to $$10^{-30} \mathrm {GeV}$$), and the ghosts are defined to have zero mass. This creates a uniform ghost density given by $$g_{t}/A_{g} $$ which is used as a proxy for the estimated pile-up contribution described by Eq. (4). These ghosts are then incorporated into the jet finding and participate in the jet clustering. By varying the amount of ghost $$p_{\text {T}}$$ density incorporated into the jet finding and determining the sensitivity of a given jet’s shape to that variation, a numerical correction can be derived. A given jet shape variable $$\mathcal{V} $$ is assumed to be a function of ghost $$p_{\text {T}}$$, $$\mathcal{V} (g_{t})$$. The reconstructed (uncorrected) jet shape is then $$\mathcal{V} (g_{t} =0)$$. The corrected jet shape can be obtained by extrapolating to the value of $$g_{t}$$ which cancels the effect of the pile-up $$p_{\text {T}}$$ density, namely $$g_{t} = -\rho \cdot A_{g} $$. The corrected shape is then given by $$\mathcal{V} _{\mathrm {corr}} = \mathcal{V} (g_{t} = -\rho \cdot A_{g})$$. This solution can be achieved by using the Taylor expansion:

7 $$\begin{aligned} \mathcal{V} _{\mathrm {corr}}= \sum \limits _{k=0}^{\infty } \left( -\rho \cdot A_{g} \right) ^{k} \frac{\partial ^k \mathcal{V} (\rho ,g_{t})}{\partial g_{t} ^k}\bigg |_{g_{t} =0}. \end{aligned}$$

The derivatives are obtained numerically by evaluating several values of $\mathcal{V} (g_{t})$ for $g_{t} \ge 0$. Only the first three terms in Eq. (7) are used for the studies presented here.

One set of shape variables which has been shown to significantly benefit from the correction defined by the expansion in Eq. (7) is the set of *N*-subjettiness observables $$\tau _N$$  [52, 53]. These observables measure the extent to which the constituents of a jet are clustered around a given number of axes denoted by *N* (typically with $$N=1,2,3$$) and are related to the corresponding subjet multiplicity of a jet. The ratios $$\tau _2/\tau _1$$ ($$\tau _{21}$$) and $$\tau _3/\tau _2$$ ($$\tau _{32}$$) can be used to provide discrimination between Standard Model jet backgrounds and boosted $$W /Z $$ bosons [31, 52, 54], top quarks [31, 52, 54, 55], or even gluinos [56]. For example, $$\tau _{21} \simeq 1$$ corresponds to a jet that is very well described by a single subjet whereas a lower value implies a jet that is much better described by two subjets rather than one.

Two approaches are tested for correcting the *N*-subjettiness ratios $\tau _{21}$ and $\tau _{32}$. The first approach is to use the individually corrected $\tau _N$ for the calculation of the numerators and denominators of the ratios. A second approach is also tested in which the full ratio is treated as a single observable and corrected directly. The resulting agreement between data and MC simulation is very similar in the two cases. However, for very high $p_{\text {T}}$ jets ($600~\mathrm {GeV}\le p_{\text {T}} ^\mathrm {jet} <800~\mathrm {GeV}$) the first approach is preferable since it yields final ratios that are closer to the values obtained for truth-particle jets and a mean $\langle \tau _{32} \rangle$ that is more stable against $\langle \mu \rangle$. On the other hand, at lower jet $p_{\text {T}}$ ($200~\mathrm {GeV}\le p_{\text {T}} ^\mathrm {jet} <300~\mathrm {GeV}$), applying the jet shape subtraction to the ratio itself performs better than the individual $\tau _N$ corrections according to the same figures of merit. Since substructure studies and the analysis of boosted hadronic objects typically focus on the high jet $p_{\text {T}}$ regime, all results shown here use the individual corrections for $\tau _N$ in order to compute the corrected $\tau _{21}$ and $\tau _{32}$.

**Fig. 8:**
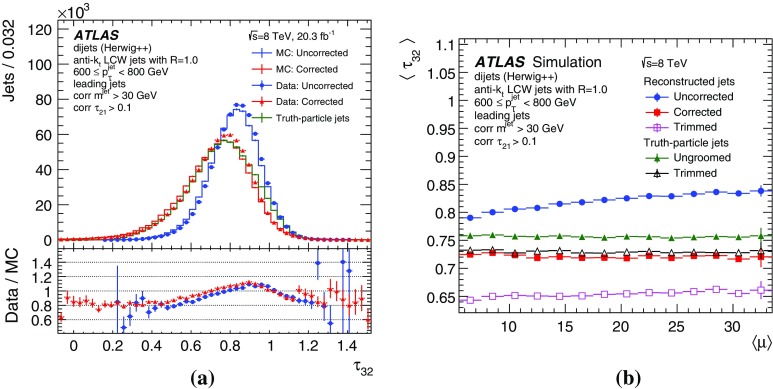
**a** Comparisons of the uncorrected (*filled blue circles*), corrected (*red*) distributions of the ratio of 3-subjettiness to 2-subjettiness ($\tau _{32}$) for data (*points*) and for MC simulation (*solid histogram*) for leading jets in the range $600 \le p_{\text {T}} < 800~\mathrm {GeV}$. The distribution of $\tau _{32}$ computed using stable truth particles (*filled green triangles*) is also included. The *lower panel* displays the ratio of the data to the MC simulation. **b** Dependence of $\tau _{32}$ on $\langle \mu \rangle$ for the uncorrected (*filled blue circles*), corrected (*filled red squares*) and trimmed (*open purple squares*) distributions for reconstructed jets in MC simulation for leading jets in the range $600 \le p_{\text {T}} < 800~\mathrm {GeV}$. The mean value of $\tau _{32}$ computed using stable truth particles (*green*) is also included

Figure 8 presents the uncorrected and corrected distributions of $\tau _{32}$, in both the observed data and MC simulation, as well as the truth-particle jet distributions. In the case of Fig. 8b, the mean value of $\tau _{32}$ is also presented for trimmed jets, using both the reconstructed and truth-particle jets. This comparison allows for a direct comparison of the shape subtraction method to trimming in terms of their relative effectiveness in reducing the pile-up dependence of the jet shape. Additional selections are applied to the jets used to study $\tau _{32}$ in this case: $\tau _{21} >0.1$ (after correction) and jet mass $m^\mathrm{jet} >30 \mathrm {GeV}$ (after correction). These selections provide protection against the case where $\tau _2$ becomes very small and small variations in $\tau _3$ can thus lead to large changes in the ratio. The requirement on $\tau _{21}$ rejects approximately 1 % of jets, whereas the mass requirement removes approximately 9 % of jets. As discussed above, the default procedure adopted here is to correct the ratio $\tau _{21}$ by correcting $\tau _1$ and $\tau _2$ separately. In cases where both the corrected $\tau _1$ and $\tau _2$ are negative, the sign of the corrected $\tau _{21}$ is set to negative.

The corrected *N*-subjettiness ratio $\tau _{32}$ shows a significant reduction in pile-up dependence, as well as a much closer agreement with the distribution expected from truth-particle jets. Figure 8b provides comparisons between the shape subtraction procedure and jet trimming. Trimming is very effective in removing the pile-up dependence of jet substructure variables (see Ref. [31] and Sect. 7). However, jet shape variables computed after jet trimming are considerably modified by the removal of soft subjets and must be directly compared to truth-level jet shape variables constructed with trimming at the truth level as well. Comparing the mean trimmed jet $\tau _{32}$ at truth level to the reconstructed quantity in Fig. 8b (open black triangles and open purple square markers, respectively), and similarly for the shape correction method (filled green triangles and filled red square markers, respectively) it is clear that the shape expansion correction obtains a mean value closer to the truth.

## Pile-up jet suppression techniques and results

The suppression of pile-up jets is a crucial component of many physics analyses in ATLAS. Pile-up jets arise from two sources: hard QCD jets originating from a pile-up vertex, and local fluctuations of pile-up activity. The pile-up QCD jets are genuine jets and must be tagged and rejected using the vertex-pointing information of charged-particle tracks (out-of-time QCD jets have very few or no associated tracks since the ID reconstructs tracks only from the in-time events). Pile-up jets originating from local fluctuations are a superposition of random combinations of particles from multiple pile-up vertices, and they are generically referred to here as *stochastic* jets. Stochastic jets are preferentially produced in regions of the calorimeter where the global $\rho$ estimate is smaller than the actual pile-up activity. Tracking information also plays a key role in tagging and rejecting stochastic jets. Since tracks can be precisely associated with specific vertices, track-based observables can provide information about the pile-up structure and vertex composition of jets within the tracking detector acceptance ($|\eta |<2.5$) that can be used for discrimination. The composition of pile-up jets depends on both $\langle \mu \rangle$ and $p_{\text {T}}$. Stochastic jets have a much steeper $p_{\text {T}}$ spectrum than pile-up QCD jets. Therefore, higher-$p_{\text {T}}$ jets that are associated with a primary vertex which is not the hard-scatter vertex are more likely to be pile-up QCD jets, not stochastic jets. On the other hand, while the number of QCD pile-up jets increases linearly with $\langle \mu \rangle$, the rate of stochastic jets increases more rapidly such that at high luminosity the majority of pile-up jets at low $p_{\text {T}}$ are expected to be stochastic in nature [57].

### Pile-up jet suppression from subtraction

**Fig. 9:**
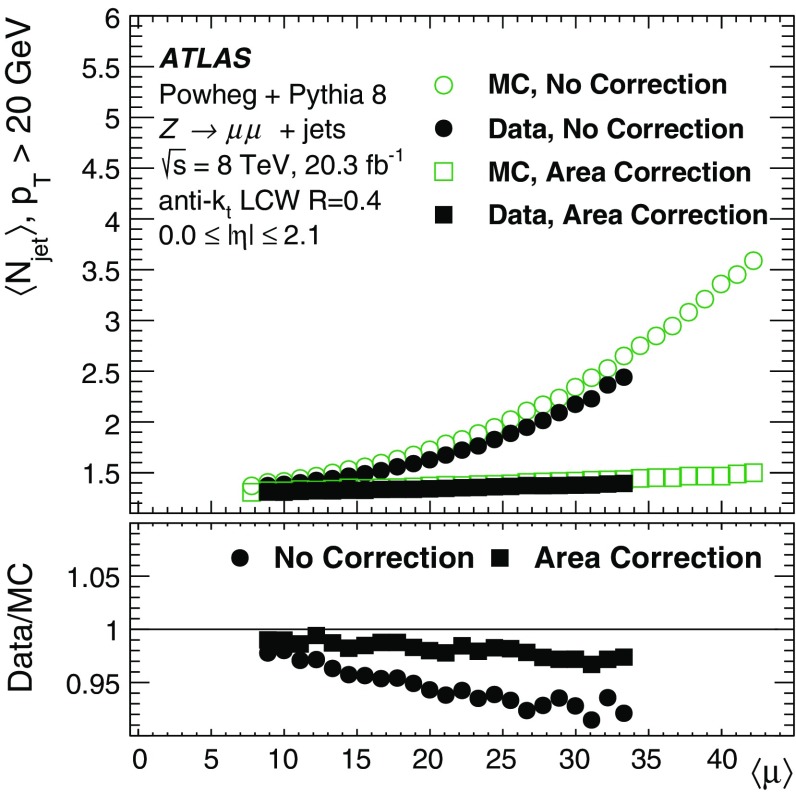
The mean anti-$$k_{t}$$ $$R=0.4$$ LCW jet multiplicity as a function of $$\langle \mu \rangle $$ in $$Z $$+jets events for jets with $$p_{\text {T}} > 20 \mathrm {GeV}$$ and $$|\eta | < 2.1$$. Events in this plot are required to have at least 1 jet both before and after the application of the jet-area based pile-up correction

The number of reconstructed jets increases with the average number of pile-up interactions, as shown in Fig. 9 using the $$Z $$+jets event sample described in Sect. 5.1. Event-by-event pile-up subtraction based on jet areas, as described in Sect. 5.2, removes the majority of pile-up jets by shifting their $$p_{\text {T}}$$ below the $$p_{\text {T}}$$ threshold of $$20~\mathrm {GeV}$$. This has the effect of improving the level of agreement between data and MC simulation. The phenomenon of pile-up jets is generally not well modelled, as shown in the ratio plot of Fig. 9.

### Pile-up jet suppression from tracking

Some pile-up jets remain even after pile-up subtraction mainly due to localised fluctuations in pile-up activity which are not fully corrected by $\rho$ in Eq. (5). Information from the tracks matched to each jet may be used to further reject any jets not originating from the hard-scatter interaction. ATLAS has developed three different track-based tagging approaches for the identification of pile-up jets: The jet vertex fraction ($\mathrm{JVF}$) algorithm, used in almost all physics analyses in Run 1, a set of two new variables ($\mathrm{corrJVF}$, and $R_\mathrm {pT}$) for improved performance, and a new combined discriminant, the jet vertex tagger ($\mathrm{JVT}$) for optimal performance. While the last two approaches were developed using Run 1 data, most analyses based on Run 1 data were completed before these new algorithms for pile-up suppression were developed. Their utility is already being demonstrated for use in high-luminosity LHC upgrade studies, and they will be available to all ATLAS analyses at the start of Run 2.

#### Jet vertex fraction

The jet vertex fraction ($\mathrm{JVF}$) is a variable used in ATLAS to identify the primary vertex from which the jet originated. A cut on the $\mathrm{JVF}$ variable can help to remove jets which are not associated with the hard-scatter primary vertex. Using tracks reconstructed from the ID information, the $\mathrm{JVF}$ variable can be defined for each jet with respect to each identified primary vertex (PV) in the event, by identifying the PV associated with each of the charged-particle tracks pointing towards the given jet. Once the hard-scatter PV is identified, the $\mathrm{JVF}$ variable can be used to select jets having a high likelihood of originating from that vertex. Tracks are assigned to calorimeter jets following the ghost-association procedure [50], which consists of assigning tracks to jets by adding tracks with infinitesimal $p_{\text {T}}$ to the jet clustering process. Then, the $\mathrm{JVF}$ is calculated as the ratio of the scalar sum of the $p_{\text {T}}$ of matched tracks that originate from a given PV to the scalar sum of $p_{\text {T}}$ of all matched tracks in the jet, independently of their origin.

$\mathrm{JVF}$ is defined for each jet with respect to each PV. For a given jet$_{i}$, its $\mathrm{JVF}$ with respect to the primary vertex PV$_{j}$ is given by:

8 $$\begin{aligned} \mathrm{JVF} (\mathrm{jet}_{i},\mathrm{PV}_{j})=\frac{\sum _m p_{\text {T}} (\mathrm{track}_{m}^\mathrm{jet_i},\mathrm{PV}_j)}{\sum _n\sum _l p_{\text {T}} (\mathrm{track}_{l}^\mathrm{jet_i},\mathrm{PV}_n)}, \end{aligned}$$

where *m* runs over all tracks originating from PV$_{j}$ 2 matched to jet$_{i}$, *n* over all primary vertices in the event and *l* over all tracks originating from PV$_{n}$ matched to jet$_{i}$. Only tracks with $p_{\text {T}} > 500~\mathrm {MeV}$ are considered in the $\mathrm{JVF}$ calculation. $\mathrm{JVF}$ is bounded by 0 and 1, but a value of $-1$ is assigned to jets with no associated tracks.

**Fig. 10:**
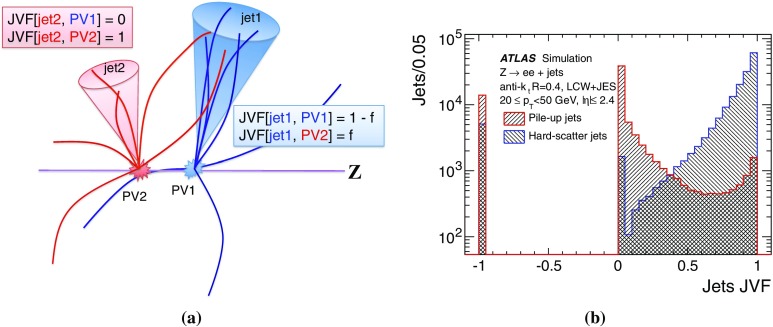
**a** Schematic representation of the jet vertex fraction $\mathrm{JVF}$ principle where *f* denotes the fraction of track $p_{\text {T}}$ contributed to jet 1 due to the second vertex (PV2). **b** $\mathrm{JVF}$ distribution for hard-scatter (*blue*) and pile-up (*red*) jets with $20< p_{\text {T}} < 50 \mathrm {GeV}$ and $|\eta |<2.4$ after pile-up subtraction and jet energy scale correction in simulated $Z+$jets events

For the purposes of this paper, $$\mathrm{JVF}$$ is defined from now on with respect to the hard-scatter primary vertex. In the $$Z $$+jets events used for these studies of pile-up suppression, this selection of the hard-scatter primary vertex is found to be correct in at least 98 % of events. $$\mathrm{JVF}$$ may then be interpreted as an estimate of the fraction of $$p_{\text {T}}$$ in the jet that can be associated with the hard-scatter interaction. The principle of the $$\mathrm{JVF}$$ variable is shown schematically in Fig. 10a. Figure 10b shows the $$\mathrm{JVF}$$ distribution in MC simulation for hard-scatter jets and for pile-up jets with $$p_{\text {T}} > 20 \mathrm {GeV}$$ after pile-up subtraction and jet energy scale correction in a $$Z(\rightarrow ee)+$$jets sample with the $$\langle \mu \rangle $$ distribution shown in Fig. 1. Hard-scatter jets are calorimeter jets that have been matched to truth-particle jets from the hard-scatter with an angular separation of $$\Delta R\le 0.4$$, whereas pile-up jets are defined as calorimeter jets with an angular separation to the nearest truth-particle jet of $$\Delta R>0.4$$. The thresholds for truth-particle jets are $$p_{T}>10$$ GeV for those originating from the hard-scatter, and $$p_{T}>4$$ GeV for those originating in pile-up interactions. This comparison demonstrates the discriminating power of the $$\mathrm{JVF}$$ variable.

While $\mathrm{JVF}$ is highly correlated with the actual fraction of hard-scatter activity in a reconstructed calorimeter jet, it is important to note that the correspondence is imperfect. For example, a jet with significant neutral pile-up contributions may receive $\mathrm{JVF} = 1$, while $\mathrm{JVF} = 0$ may result from a fluctuation in the fragmentation of a hard-scatter jet such that its charged constituents all fall below the track $p_{\text {T}}$ threshold. $\mathrm{JVF}$ also relies on the hard-scatter vertex being well separated along the beam axis from all of the pile-up vertices. In some events, a pile-up jet may receive a high value of $\mathrm{JVF}$ because its associated primary vertex is very close to the hard-scatter primary vertex. While this effect is very small for 2012 pile-up conditions, it will become more important at higher luminosities, as the average distance between interactions decreases as $1/\langle \mu \rangle$. For these reasons, as well as the lower probability for producing a pile-up QCD jet at high $p_{\text {T}}$, $\mathrm{JVF}$ selections are only applied to jets with $p_{\text {T}} \le 50~\mathrm {GeV}$.

**Fig. 11:**
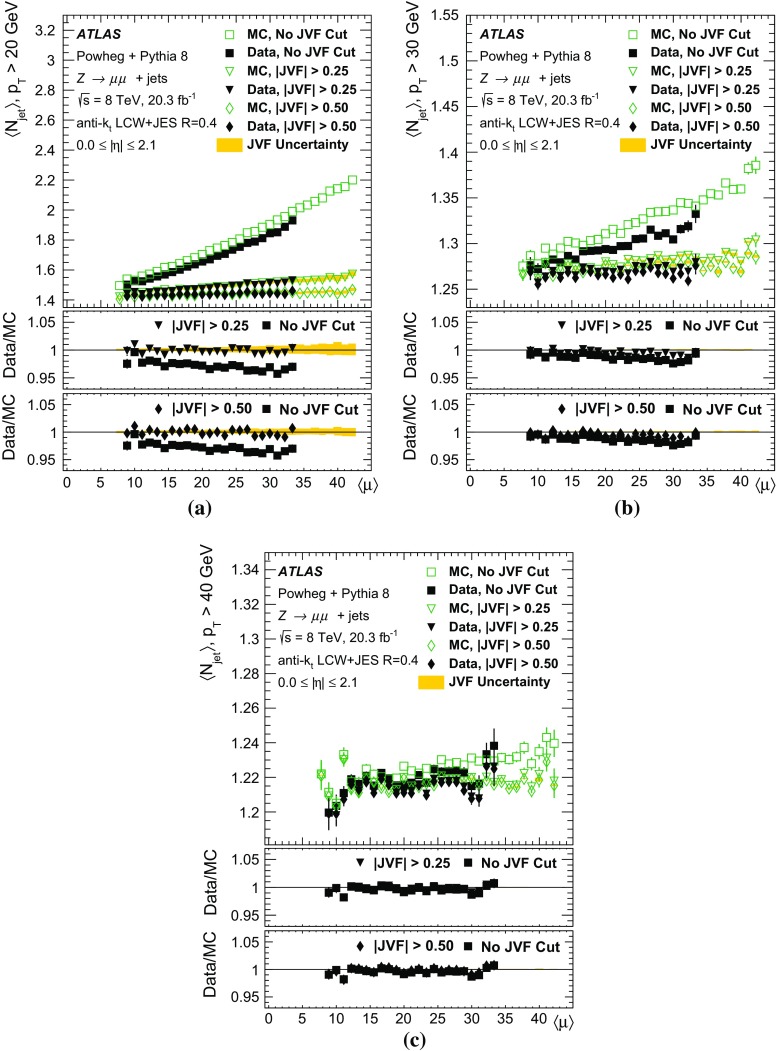
The mean anti-$$k_{t}$$ $$R=0.4$$ LCW+JES jet multiplicity as a function of $$\langle \mu \rangle $$ in $$Z $$+jets events for jets with $$|\eta | < 2.1$$, back-to-back with the $$Z$$ boson, before and after several $$|\mathrm{JVF} |$$ cuts were applied to jets with $$p_{\text {T}} < 50~\mathrm {GeV}$$. Results for jets with **a** $$p_{\text {T}} > 20~\mathrm {GeV}$$, **b** $$p_{\text {T}} > 30~\mathrm {GeV}$$ and **c** $$p_{\text {T}} > 40~\mathrm {GeV}$$ are shown requiring at least one jet of that $$p_{\text {T}}$$. To remove effects of hard-scatter modelling the dependence on $$\langle \mu \rangle $$ was fit and the MC simulation shifted so that data and simulation agree at zero pile-up, $$\langle \mu \rangle = 0$$. The *upper ratio plots* show results before and after applying a $$|\mathrm{JVF} |$$ cut of 0.25 and the *lower ratio plots* show the same for a cut of 0.50. The $$\mathrm{JVF}$$ uncertainty is very small when counting jets with $$p_{\text {T}} > 40~\mathrm {GeV}$$

The modelling of $\mathrm{JVF}$ is investigated in $Z (\rightarrow \mu \mu )$+jets events using the same selection as discussed in Sect. 5.1, which yields a nearly pure sample of hard-scatter jets. By comparison to truth-particle jets in MC simulation, it was found that the level of pile-up jet contamination in this sample is close to 2 % near $20~\mathrm {GeV}$ and almost zero at the higher end of the range near $50~\mathrm {GeV}$. The $\mathrm{JVF}$ distribution for the jet balanced against the *Z* boson in these events is well modelled for hard-scatter jets. However, the total jet multiplicity in these events is overestimated in simulated events, due to mismodelling of pile-up jets. This is shown in Fig. 11, for several different choices of the minimum $p_{\text {T}}$ cut applied at the fully calibrated jet energy scale (including jet-area-based pile-up subtraction). The application of a $\mathrm{JVF}$ cut significantly improves the data/MC agreement because the majority of pile-up jets fail the $\mathrm{JVF}$ cut: across all $p_{\text {T}}$ bins, data and MC simulation are seen to agree within 1 % following the application of a $\mathrm{JVF}$ cut. It is also observed that the application of a $\mathrm{JVF}$ cut results in stable values for the mean jet multiplicity as a function of $\langle \mu \rangle$.

Figure 11 also shows the systematic uncertainty bands, which are only visible for the lowest $p_{\text {T}}$ selection of $20~\mathrm {GeV}$. These uncertainties are estimated by comparing the $\mathrm{JVF}$ distributions for hard-scatter jets in data and MC simulation. The efficiency of a nominal $\mathrm{JVF}$ cut of *X* is defined as the fraction of jets, well balanced against the *Z* boson, passing the cut, denoted by $\mathcal {E}_\mathrm{MC}^\mathrm{nom}$ and $\mathcal {E}_\mathrm{data}^\mathrm{nom}$ for MC events and data, respectively. The systematic uncertainty is derived by finding two $\mathrm{JVF}$ cuts with $\mathcal {E}_\mathrm{MC}$ differing from $\mathcal {E}_\mathrm{MC}^\mathrm{nom}$ by $\pm (\mathcal {E}_\mathrm{MC}^\mathrm{nom}-\mathcal {E}_\mathrm{data}^\mathrm{nom})$. The $\mathrm{JVF}$ uncertainty band is then formed by re-running the analysis with these up and down variations in the $\mathrm{JVF}$ cut value. Systematic uncertainties vary between 2 and 6 % depending on jet $p_{\text {T}}$ and $\eta$.

#### Improved variables for pile-up jet vertex identification

While a $\mathrm{JVF}$ selection is very effective in rejecting pile-up jets, it has limitations when used in higher (or varying) luminosity conditions. As the denominator of $\mathrm{JVF}$ increases with the number of reconstructed primary vertices in the event, the mean $\mathrm{JVF}$ for signal jets is shifted to smaller values. This explicit pile-up dependence of $\mathrm{JVF}$ results in an $N_{\mathrm{PV}}$-dependent jet efficiency when a minimum $\mathrm{JVF}$ criterion is imposed to reject pile-up jets. This pile-up sensitivity is addressed in two different ways. First, by correcting $\mathrm{JVF}$ for the explicit pile-up dependence in its denominator ($\mathrm{corrJVF}$) and, second, by introducing a new variable defined entirely from hard-scatter observables ($R_\mathrm {pT}$).

The quantity $\mathrm{corrJVF}$ is a variable similar to $\mathrm{JVF}$, but corrected for the $N_{\mathrm{PV}}$ dependence. It is defined as

9 $$\begin{aligned} \mathrm{corrJVF} = \frac{\sum _m{p_{\mathrm{T}, m}^{\mathrm{track}}(\mathrm{PV}_0)}}{\sum _l p_{\mathrm{T}, l}^{\mathrm{track}}(\mathrm{PV}_0) + \frac{\sum _{n\ge 1}\sum _l p_{\mathrm{T}, l}^{\mathrm{track}}(\mathrm{PV}_n)}{ (k \cdot n_\mathrm {track}^\mathrm {PU})}}, \end{aligned}$$

where $\sum _m{p_{\mathrm{T}, m}^{\mathrm{track}}(\mathrm{PV}_0)}$ is the scalar sum of the $p_{\text {T}}$ of the tracks that are associated with the jet and originate from the hard-scatter vertex. The term $\sum _{n\ge 1}\sum _l p_{\mathrm{T}, l}^{\mathrm{track}}(\mathrm{PV}_n)=p_{\text {T}} ^{\mathrm {PU}}$ denotes the scalar sum of the $p_{\text {T}}$ of the associated tracks that originate from any of the pile-up interactions.

The $\mathrm{corrJVF}$ variable uses a modified track-to-vertex association method that is different from the one used for $\mathrm{JVF}$. The new selection aims to improve the efficiency for *b*-quark jets and consists of two steps. In the first step, the vertex reconstruction is used to assign tracks to vertices. If a track is attached to more than one vertex, priority is given to the vertex with higher $\sum (p_{\text {T}} ^{\mathrm {track}})^2$. In the second step, if a track is not associated with any primary vertex after the first step but satisfies $|\Delta z|<3$ mm with respect to the hard-scatter primary vertex, it is assigned to the hard-scatter primary vertex. The second step targets tracks from decays in flight of hadrons that originate from the hard-scatter but are not likely to be attached to any vertex. The $|\Delta z|<3$ mm criterion was chosen based on the longitudinal impact parameter distribution of tracks from *b*-hadron decays, but no strong dependence of the performance on this particular criterion was observed when the cut value was altered within 1 mm. The new 2-step track-to-vertex association method results in a significant increase in the hard-scatter jet efficiency at fixed rate of fake pile-up jets, with a large performance gain for jets initiated by *b*-quarks.

To correct for the linear increase of $\langle p_{\text {T}} ^{\mathrm {PU}}\rangle$ with the total number of pile-up tracks per event ($n_\mathrm {track}^\mathrm {PU}$), $p_{\text {T}} ^{\mathrm {PU}}$ is divided by $(k \cdot n_\mathrm {track}^\mathrm {PU})$, with $k=0.01$, in the $\mathrm{corrJVF}$ definition. The total number of pile-up tracks per event is computed from all tracks associated with vertices other than the hard-scatter vertex. The scaling factor *k* is approximated by the slope of $\langle p_{\text {T}} ^{\mathrm {PU}}\rangle$ with $n_\mathrm {track}^\mathrm {PU}$, but the resulting discrimination between hard-scatter and pile-up jets is insensitive to the choice of *k*.^3^

**Fig. 12:**
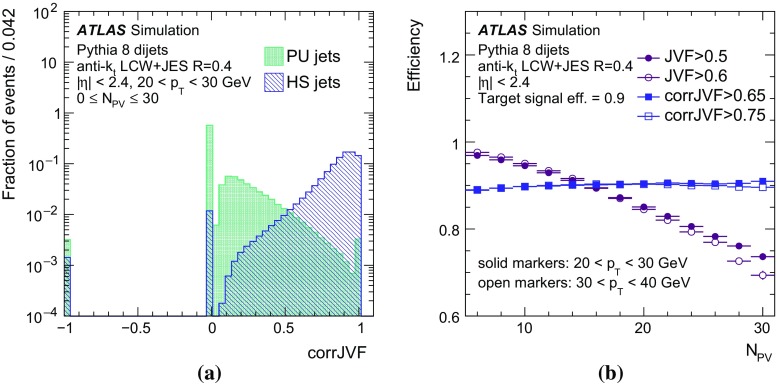
**a** Distribution of $\mathrm{corrJVF}$ for pile-up (PU) and hard-scatter (HS) jets with $20< p_{\text {T}} < 30~\mathrm {GeV}$. **b** Primary-vertex dependence of the hard-scatter jet efficiency for $20< p_{\text {T}} < 30~\mathrm {GeV}$ (*solid markers*) and $30< p_{\text {T}} < 40~\mathrm {GeV}$ (*open markers*) jets for fixed cuts of $\mathrm{corrJVF}$ (*blue square*) and $\mathrm{JVF}$ (*violet circle*) such that the inclusive efficiency is $90~\%$. The selections placed on $\mathrm{corrJVF}$ and $\mathrm{JVF}$, which depend on the $p_{\text {T}}$ bin, are specified in the legend

Figure 12a shows the $\mathrm{corrJVF}$ distribution for pile-up and hard-scatter jets in simulated dijet events. A value $\mathrm{corrJVF} =-1$ is assigned to jets with no associated tracks. Jets with $\mathrm{corrJVF} = 1$ are not included in the studies that follow due to use of signed $\mathrm{corrJVF}$ selections. About $1~\%$ of hard-scatter jets with $20< p_{\text {T}} < 30~\mathrm {GeV}$ have no associated hard-scatter tracks and thus $\mathrm{corrJVF} =0$.

Figure 12b shows the hard-scatter jet efficiency as a function of the number of reconstructed primary vertices in the event when imposing a minimum $\mathrm{corrJVF}$ or $\mathrm{JVF}$ requirement such that the efficiency measured across the full range of $N_{\mathrm{PV}}$ is 90 %. For the full range of $N_{\mathrm{PV}}$ considered, the hard-scatter jet efficiency after a selection based on $\mathrm{corrJVF}$ is stable at $90\%\pm 1\%$, whereas for $\mathrm{JVF}$ the efficiency degrades by about 20 %, from 97 to 75 %. The choice of scaling factor *k* in the $\mathrm{corrJVF}$ distribution does not affect the stability of the hard-scatter jet efficiency with $N_{\mathrm{PV}}$.

The variable $R_\mathrm {pT}$ is defined as the scalar sum of the $p_{\text {T}}$ of the tracks that are associated with the jet and originate from the hard-scatter vertex divided by the fully calibrated jet $p_{\text {T}}$, which includes pile-up subtraction:

10 $$\begin{aligned} R_\mathrm {pT} = \frac{\sum _k{p_{\mathrm{T}, k}^{\mathrm{track}}(\mathrm{PV}_0)}}{p_{\text {T}} ^\mathrm {jet}}. \end{aligned}$$

**Fig. 13:**
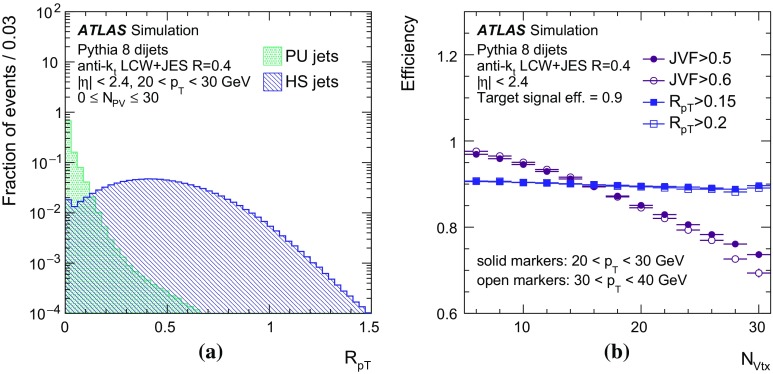
**a** Distribution of $R_\mathrm {pT}$ for pile-up (PU) and hard-scatter (HS) jets with $20< p_{\text {T}} < 30~\mathrm {GeV}$. **b** Primary-vertex dependence of the hard-scatter jet efficiency for $20< p_{\text {T}} < 30\mathrm {GeV}$ (*solid markers*) and $30< p_{\text {T}} < 40~\mathrm {GeV}$ (*open markers*) jets for fixed cuts of $R_\mathrm {pT}$ (*blue square*) and $\mathrm{JVF}$ (*violet circle*) such that the inclusive efficiency is $90~\%$. The cut values imposed on $R_\mathrm {pT}$ and $\mathrm{JVF}$, which depend on the $p_{\text {T}}$ bin, are specified in the legend

The $R_\mathrm {pT}$ distributions for pile-up and hard-scatter jets are shown in Fig. 13a. $R_\mathrm {pT}$ is peaked at 0 and is steeply falling for pile-up jets, since tracks from the hard-scatter vertex rarely contribute. For hard-scatter jets, however, $R_\mathrm {pT}$ has the meaning of a charged $p_{\text {T}}$ fraction and its mean value and spread are larger than for pile-up jets. Since $R_\mathrm {pT}$ involves only tracks that are associated with the hard-scatter vertex, its definition is at first order independent of $N_{\mathrm{PV}}$. Figure 13b shows the hard-scatter jet efficiency as a function of $N_{\mathrm{PV}}$ when imposing a minimum $R_\mathrm {pT}$ and $\mathrm{JVF}$ requirement such that the $N_{\mathrm{PV}}$ inclusive efficiency is $90~\%$. For the full range of $N_{\mathrm{PV}}$ considered, the hard-scatter jet efficiency after a selection based on $R_\mathrm {pT}$ is stable at $90~\%\pm 1~\%$.

#### Jet vertex tagger

A new discriminant called the jet vertex tagger ($\mathrm{JVT}$) is constructed using $R_\mathrm {pT}$ and $\mathrm{corrJVF}$ as a two-dimensional likelihood derived using simulated dijet events and based on a k-nearest neighbour (kNN) algorithm [58]. For each point in the two-dimensional $\mathrm{corrJVF}-R_\mathrm {pT}$ plane, the relative probability for a jet at that point to be of signal type is computed as the ratio of the number of hard-scatter jets to the number of hard-scatter plus pile-up jets found in a local neighbourhood around the point using a training sample of signal and pile-up jets with $20< p_{\text {T}} < 50~\mathrm {GeV}$ and $|\eta | < 2.4$. The local neighbourhood is defined dynamically as the 100 nearest neighbours around the test point using a Euclidean metric in the $R_\mathrm {pT}$–$\mathrm{corrJVF}$ space, where $\mathrm{corrJVF}$ and $R_\mathrm {pT}$ are rescaled so that the variables have the same range.

**Fig. 14:**
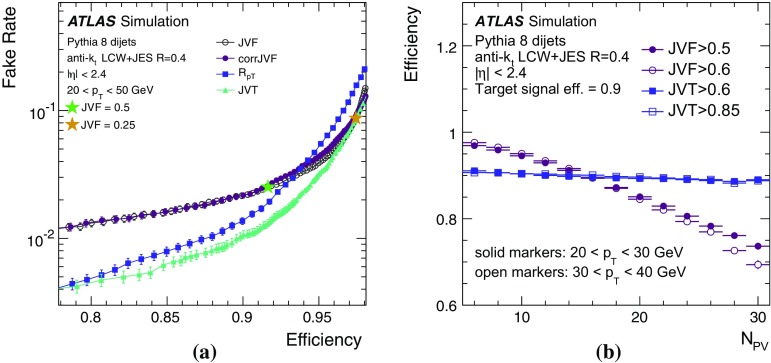
**a** Fake rate from pile-up jets versus hard-scatter jet efficiency curves for $\mathrm{JVF}$, $\mathrm{corrJVF}$, $R_\mathrm {pT}$ and $\mathrm{JVT}$. The widely used $\mathrm{JVF}$ working points with cut values 0.25 and 0.5 are indicated with *gold* and *green stars*. **b** Primary vertex dependence of the hard-scatter jet efficiency for $20< p_{\text {T}} < 30~\mathrm {GeV}$ (*solid markers*) and $30< p_{\text {T}} < 40~\mathrm {GeV}$ (*open markers*) jets for fixed cuts of $\mathrm{JVT}$ (*blue square*) and $\mathrm{JVF}$ (*violet circle*) such that the inclusive efficiency is $90~\%$

Figure 14a shows the fake rate versus efficiency curves comparing the performance of the four variables $\mathrm{JVF}$, $\mathrm{corrJVF}$, $R_\mathrm {pT}$ and $\mathrm{JVT}$ when selecting a sample of jets with $20< p_{\text {T}} < 50~\mathrm {GeV}$, $|\eta |<2.4$ in simulated dijet events.

The figure shows the fraction of pile-up jets passing a minimum $\mathrm{JVF}$, $\mathrm{corrJVF}$, $R_\mathrm {pT}$ or $\mathrm{JVT}$ requirement as a function of the signal-jet efficiency resulting from the same requirement. The $\mathrm{JVT}$ performance is driven by $\mathrm{corrJVF}$ ($R_\mathrm {pT}$) in the region of high signal-jet efficiency (high pile-up rejection). Using $\mathrm{JVT}$, signal jet efficiencies of 80, 90 and $95~\%$ are achieved for pile-up fake rates of respectively 0.4, 1.0 and $3~\%$. When imposing cuts on $\mathrm{JVF}$ that result in the same jet efficiencies, the pile-up fake rates are 1.3, 2.2 and $4~\%$.

The dependence of the hard-scatter jet efficiencies on $N_{\mathrm{PV}}$ is shown in Fig. 14b. For the full range of $N_{\mathrm{PV}}$ considered, the hard-scatter jet efficiencies after a selection based on $\mathrm{JVT}$ are stable within $1~\%$.

The differences in fragmentation and showering between jets initiated by gluons and light quarks affect the shapes of the $\mathrm{corrJVF}$ and $R_\mathrm {pT}$ distributions and thus the performance of the $\mathrm{JVT}$-based pile-up jet suppression. Jets initiated by light quarks (*u*, *d*, *s*) have on average a lower number of associated hard-scatter tracks but a slightly higher jet energy response [59] and both effects lead towards an increase in the number of jets with no associated tracks from the hard-scatter primary vertex relative to gluon-initiated jets.

**Fig. 15:**
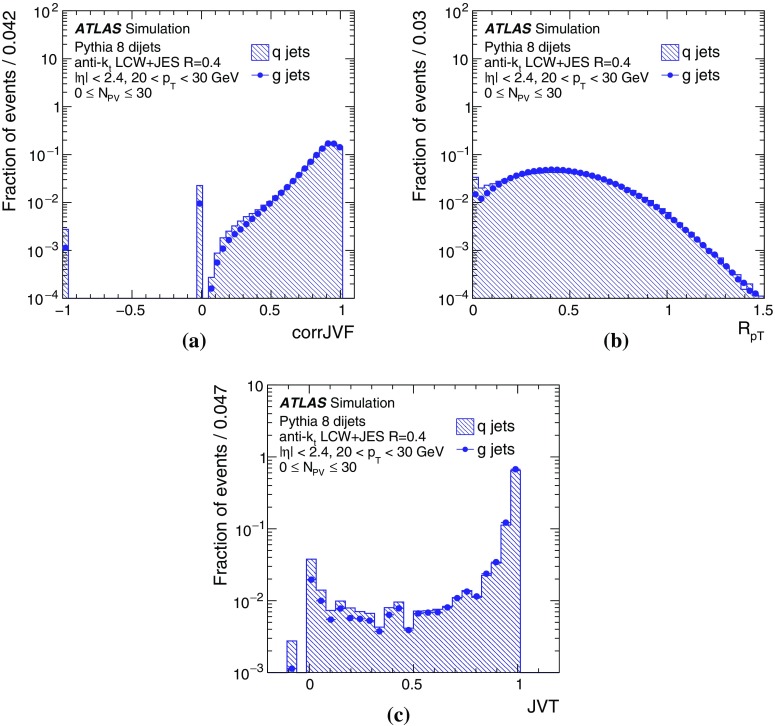
The distributions of **a** $\mathrm{corrJVF}$, **b** $R_\mathrm {pT}$ and **c** $\mathrm{JVT}$ for light-quark and gluon initiated hard-scatter jets

Figure 15 shows the $\mathrm{corrJVF}$, $R_\mathrm {pT}$ and $\mathrm{JVT}$ distributions for hard-scatter jets with $20<p_{\text {T}} <30~\mathrm {GeV}$ initiated by gluons and light quarks. Using a leading-order notion of jet flavour, the parton-level flavour labelling refers to the highest-energy parton within a narrow cone of size $\Delta R = 0.3$ around the jet axis. The distributions for jets initiated by light quarks have more entries at low $\mathrm{corrJVF}$, $R_\mathrm {pT}$ and $\mathrm{JVT}$ values and consequently a worse separation from pile-up jets. Most notably, about twice as many light-quark jets have no associated tracks from the hard-scatter primary vertex, that is $\mathrm{corrJVF} =\mathrm{JVT} =0$.

**Fig. 16:**
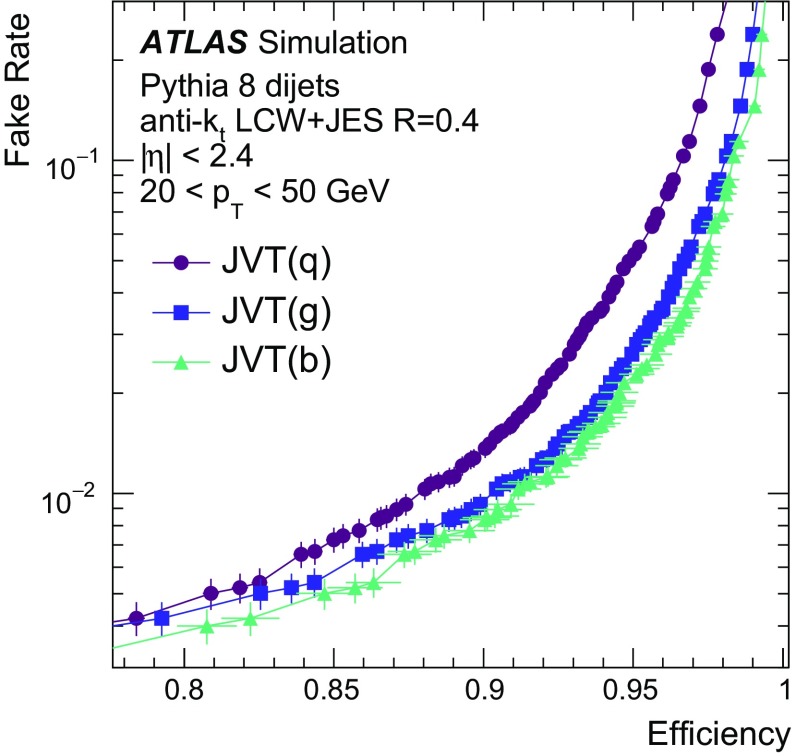
The fake rate from pile-up jets versus hard-scatter jet efficiency curves for $\mathrm{JVT}$ separating jets initiated by light quarks, gluons and *b*-quarks

Figure 16 shows the efficiency versus fake-rate curve for $\mathrm{JVT}$ for jets initiated by light quarks, gluons and *b*-quarks. As expected from Fig. 15, the performance is worse for jets initiated by light quarks. The pile-up versus hard-scatter jet discrimination performs best for hard-scatter jets initiated by *b*-quarks. The efficiency versus fake-rate curve for jets initiated by *c*-quarks is similar to that of gluon jets.

The stability of the hard-scatter efficiencies as a function of $N_{\mathrm{PV}}$ is found to be independent of the flavour of the parton initiating the jet.

To test the sample dependence of $\mathrm{JVT}$, the likelihood was also derived using a sample of $20< p_{\text {T}} < 50~\mathrm {GeV}$ jets in simulated $Z (\rightarrow \mu \mu )$+jets events. The performance of the $\mathrm{JVT}$-based pile-up jet suppression (evaluated in terms of fake rate versus efficiency curves) was found to be independent of the sample from which the likelihood is derived.

The hard-scatter jet efficiency for $\mathrm{JVT}$ in data was measured using the tag-and-probe method in $Z (\rightarrow \mu \mu )$+jets events, using a procedure similar to that described in Sect. 6.2.1 (see also Ref. [60]). Using the leading jet recoiling against the $Z$ boson as a probe, a signal region for hard-scatter jets is defined as the back-to-back region specified by the requirement $|\Delta \phi (Z , \mathrm{jet})| > 2.8$. The pile-up contamination in the signal region is estimated from a pile-up control region, based on the assumption that the $|\Delta \phi (Z , \mathrm{jet})|$ distribution is flat for pile-up jets.

**Fig. 17:**
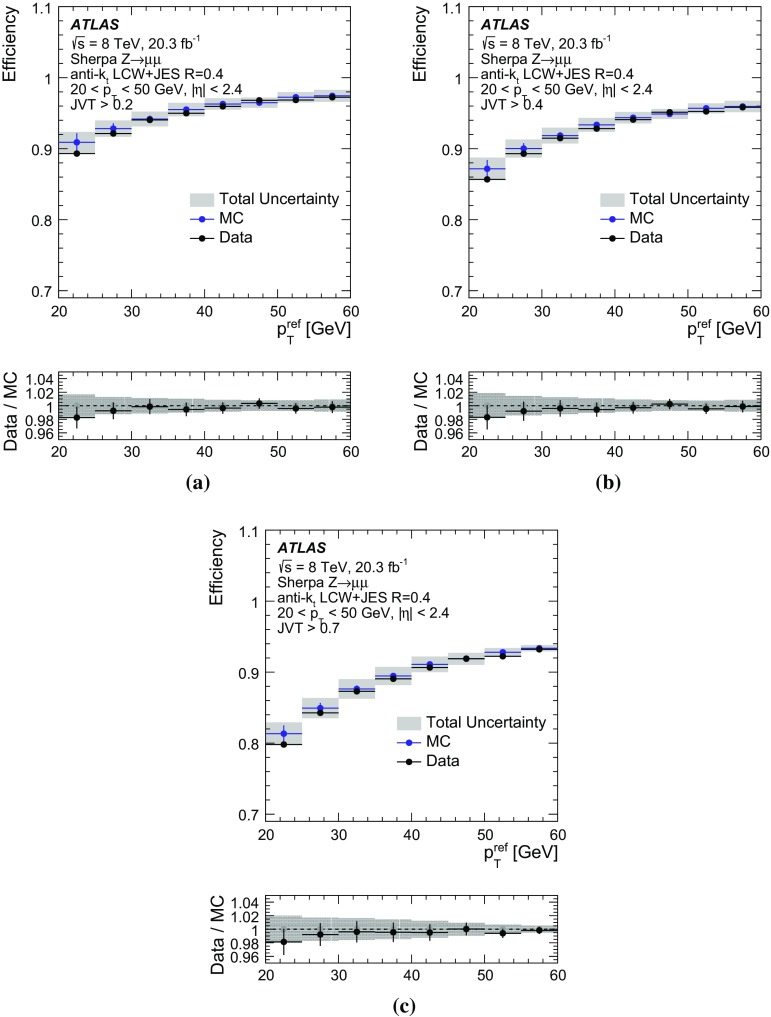
Efficiency measured in $Z (\rightarrow \mu \mu )$+jets events as a function of $p_{\text {T}} ^{\mathrm {ref}}$ in data and MC simulation for **a** $\mathrm{JVT} >0.2$, **b** $\mathrm{JVT} >0.4$ and **c** $\mathrm{JVT} >0.7$, where $p_{\text {T}} ^{\mathrm {ref}} = p_{\text {T}} ^{Z}$. The *bottom panels* of each figure show the ratio of efficiencies measured in data and MC simulation

Figure 17a, b show the jet efficiencies for minimum $\mathrm{JVT}$ requirements of 0.2, 0.4 and 0.7 respectively, measured in bins of $p_{\text {T}} ^{\mathrm {ref}} = p_{\text {T}} ^{Z}$.

Good agreement is observed between data and simulation, although there is a very slight tendency for the MC simulation to predict an efficiency higher than that found in data at low $p_{\text {T}} ^{\mathrm {ref}}$, but this difference is within the statistical uncertainty. The simulation-to-data scale factors are consistent with unity within the uncertainties. The grey band reflects the total uncertainty on the efficiency in simulation, adding the statistical and the systematic uncertainties in quadrature. The systematic uncertainty is determined by accounting for the differences in efficiency observed between the SHERPA and the POWHEG+PYTHIA  8 $Z (\rightarrow \mu \mu )$+jets MC samples, and by the mismodelling in the simulation of the $\Delta \phi (Z , \mathrm{jet})$ shape for hard-scatter jets. The total uncertainty ranges from 2 to 1 % when $p_{\text {T}} ^{\mathrm {ref}}$ varies from 20 to $60~\mathrm {GeV}$.

## Jet grooming for pile-up mitigation and suppression

The algorithmic removal of substructures within a jet based on kinematic criteria is generally referred to as *jet grooming*. Several types of jet grooming have been explored in ATLAS [31] for their ability to reduce the backgrounds to boosted-object selection while maintaining high efficiencies for signal processes. Improving the individual jet mass resolution and mitigating the effects of pile-up are critical issues in these studies. Indeed, these measures of performance are used as some of the primary figures of merit in determining a subset of groomed-jet algorithms on which to focus for physics analysis in ATLAS.

Previous studies show that trimming and filtering both significantly reduce the dependence of the jet mass on pile-up  [31]. As described in Sect. 3.1, trimming removes subjets with $p_{\mathrm{T}i}/p_{\text {T}} ^\mathrm {jet} < f_\mathrm{cut}$, where $p_{\mathrm{T}i}$ is the transverse momentum of the *i*th subjet and $f_\mathrm{cut} =0.05$. Filtering proceeds similarly, but it utilises the relative masses of the subjets defined and the original jet. For at least one of the configurations tested, trimming and filtering are both able to approximately eliminate the pile-up dependence of the jet mass. Building upon the success of calorimeter-based grooming methods and track-based pile-up suppression of small-radius jets, a new, track-based, grooming technique can be designed by vetoing individual subjets of large-$R$ jets that are associated with pile-up interactions using tracking information.

The implementations of track-based grooming in ATLAS have so far focused on $\mathrm{corrJVF}$ and so-called *jet cleansing* methods [61]. The algorithm which uses $\mathrm{corrJVF}$ relies on the application of $\mathrm{corrJVF}$ to the individual subjets of large-$R$ jets wherein tracks matched to each subjet are used in the calculation of $\mathrm{corrJVF}$ for that subjet. In particular, track-based trimming is implemented by replacing the $f_\mathrm{cut}$ criterion with a requirement on the $\mathrm{corrJVF}$ of subjets.

**Fig. 18:**
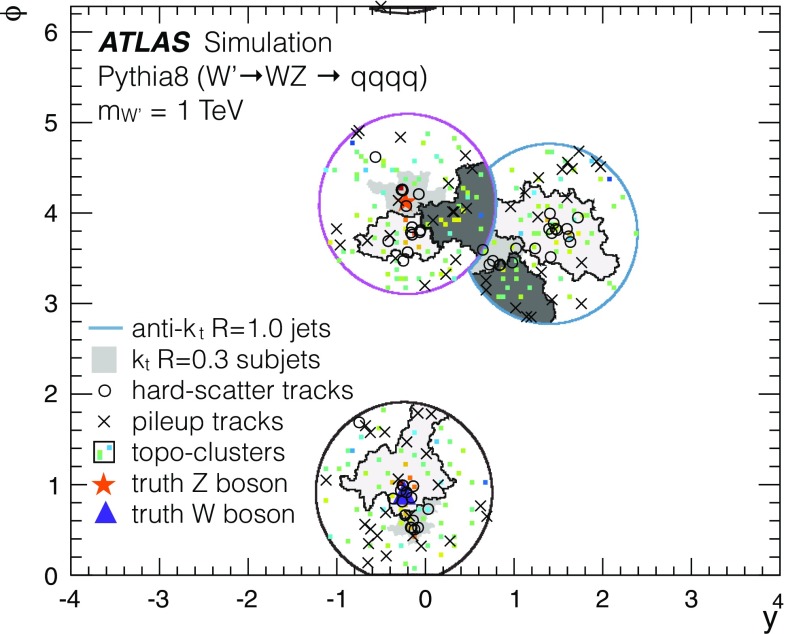
Rapidity–$\phi$ view of a simulated event of a $W ^{\prime }$ boson with a mass of 1 TeV decaying to a $W$ boson and a $Z$ boson, both of which decay to jet pairs

The concept of track-based grooming can be illustrated in an event display. Figure 18 shows both calorimeter and tracking information in the rapidity (*y*) versus azimuthal angle ($\phi$) plane of a simulated event where a $W ^{\prime }$ boson with a mass of 1 TeV decays to a $W$ boson and a $Z$ boson, which decay hadronically. The orange star and blue triangle indicate the *y*–$\phi$ positions of the generated $W$ and $Z$ bosons. The large circles represent the active area boundaries of the anti-$k_{t}$ $R=1.0$ jets, built from topological clusters. In the following, these jets are referred to as ungroomed jets. The clusters are represented by small solid squares with colours ranging from blue to red encoding low to high transverse energies. The grey regions indicate the active areas of the $k_{t}$ $R=0.3$ subjets reconstructed from the constituents of the ungroomed jets. Only subjets with a $p_{\text {T}}$ of at least $5~\%$ of the ungroomed jet $p_{\text {T}}$ are shown. Tracks associated with the jet and originating from the hard-scatter vertex (black open circles) or from pile-up vertices (black crosses) are also indicated. The violet ungroomed jet (with $\phi \approx 4.1$ and $y \approx -0.2$) has a $p_{\text {T}}$ of $446~\mathrm {GeV}$ and is matched in $\Delta R$ to the truth $Z$ boson. While all three subjets have active areas overlapping with the *y*–$\phi$ positions of pile-up tracks, only two subjets have associated hard-scatter tracks. The invariant mass reconstructed from the two subjets with hard-scatter tracks is $89~\mathrm {GeV}$ and the one from all three subjets is $119~\mathrm {GeV}$. This event display shows that tracking information can provide information complementary to calorimeter-based trimming. Track-assisted trimming would allow the rejection of the third subjet, which is likely to originate from pile-up, while keeping the two subjets from the $Z$ boson.

**Fig. 19:**
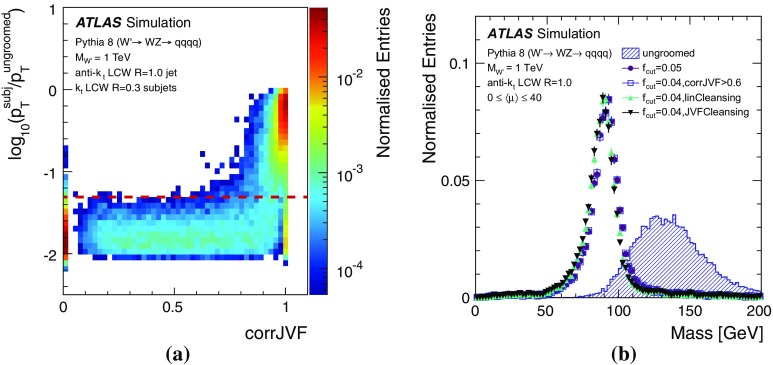
**a** Correlation of subjet $p_{\text {T}}$ fraction, defined as the ratio of the subjet $p_{\text {T}}$ to the ungroomed jet $p_{\text {T}}$, and subjet $\mathrm{corrJVF}$ for anti-$k_{t}$ $R=1.0$ jets with $p_{\text {T}} >300~\mathrm {GeV}$ and $|\eta | <1.5$. The *dotted line* indicates the standard calorimeter-based trimming $f_\mathrm{cut}$ of $5~\%$. **b** Distribution of jet mass for calorimeter- and track-based trimming configurations and jet cleansing. The default trimmed jet mass (*purple filled circles*) with $f_\mathrm{cut} = 0.05$ is compared to calorimeter-based trimming with $(f_\mathrm{cut} = 0.04)$ and $\mathrm{corrJVF} >0.6$ (*blue open squares*), linear cleansing (*green upward triangles*) and $\mathrm{JVF}$ cleansing (*black downward triangles*). The *dashed blue histogram* is the mass distribution for ungroomed jets, with no pile-up subtraction applied

Figure 19a shows the ratio of the subjet $p_{\text {T}}$ to the ungroomed jet $p_{\text {T}}$ on a logarithmic scale as a function of the subjet $\mathrm{corrJVF}$ in simulated $W^{\prime } \rightarrow W Z \rightarrow qqqq$ events. The subjet $p_{\text {T}}$ is defined as the four-momentum sum of the constituents contained within the $k_{t}$ jet that forms the subjet. The ungroomed jet $p_{\text {T}}$ is defined as the $p_{\text {T}}$ of the large-$R$ jet from which the subjets are then constructed. The two-dimensional distribution of this ratio is normalised to unit area. Approximately 4 % of subjets have no associated tracks ($\mathrm{corrJVF} =-1$) and are omitted. Most subjets with significant $p_{\text {T}}$ ratio also have large $\mathrm{corrJVF}$, indicating that most of their charged $p_{\text {T}}$ comes from the hard-scatter vertex. A large fraction of subjets with a low $p_{\text {T}}$ ratio $<5~\%$ $(\log _{10}[p_{\text {T}} ^\mathrm{sub}/p_{\text {T}} ^\mathrm{ungroomed}] < -1.3)$ and a few subjets with a significant $p_{\text {T}}$ ratio, however, have small $\mathrm{corrJVF}$ values. Most such subjets are consistent with pile-up and are excluded by the track-based jet grooming procedure. Similarly, subjets with small $p_{\text {T}}$ ratio and large $\mathrm{corrJVF}$ that would be removed by calorimeter-based trimming, are kept by the track-based trimming algorithm.

For the 2012 pile-up conditions with an average of about 21 $pp$ interactions per bunch crossing, an $f_\mathrm{cut}$ of 4 % in addition to the requirement of $\mathrm{corrJVF} >0.6$ is found to be the optimal combination of trimming and $\mathrm{corrJVF}$ selection. A grooming configuration based solely on $\mathrm{corrJVF}$ (with no $f_\mathrm{cut}$ applied) is found to have a slightly worse mass resolution than trimming alone.

The jet cleansing approach is implemented in two forms: $\mathrm{JVF}$ cleansing and linear cleansing. In $\mathrm{JVF}$ cleansing, the four-momentum of each subjet is scaled by the subjet $\mathrm{JVF}$, aiming to approximate the momentum of the subjet arising from neutral and charged particles from the hard-scatter vertex only. In linear cleansing, the subjet four-momentum from the hard-scatter vertex is approximated by scaling the reconstructed four-momentum based on the assumption that the ratio of charged to charged plus neutral pile-up $p_{\text {T}}$ contributing to a subjet is 0.55 [61]. Each $f_\mathrm{cut}$ used in these procedures is chosen to optimise the mass resolution. For 2012 pile-up conditions, the application of track-assisted grooming achieves a similar mass resolution to that of calorimeter-based trimming.

Figure 19b compares the performance of the track-assisted grooming procedure with the variants of the jet cleansing concept. All of the methods studied show significant improvements in the jet mass resolution and stability with respect to pile-up. For the pile-up conditions expected during the LHC Run 1 and Run 2, studies using simulated data do not exhibit any significant difference between $\mathrm{corrJVF}$ and jet cleansing. However, for higher luminosity conditions expected beyond 2023 at the LHC the track-based grooming provides an alternative to calorimeter-only approaches. Another advantage of track-based grooming over standard calorimeter-based grooming is that no $p_{\text {T}}$ threshold is involved in the removal of subjets. This means that in the limit of no pile-up, track-based grooming does not remove any signal, unlike for example trimming, which always rejects subjets that fall below the $f_\mathrm{cut}$ threshold.

## Conclusions

The presence of multiple simultaneous proton-proton interactions, known as pile-up, is one of the major challenges for jet reconstruction at the LHC. ATLAS has implemented three main techniques to mitigate the effect of pile-up on jets and jet measurements: topological clustering, event-by-event jet pile-up subtraction, and jet vertex tagging pile-up jet suppression. The first method reduces the impact of pile-up at the constituent level, whereas the latter two techniques are applied after jet reconstruction, to correct jet kinematic and substructure variables and to suppress jets induced by pile-up.

Topological clustering partially suppresses the formation of calorimeter clusters from pile-up activity, before jet reconstruction, by considering pile-up as a form of noise in the definition of the energy significance thresholds for cells. This acts as a constituent-level pile-up suppression and significantly reduces the contribution of pile-up to the inputs to jet reconstruction. For the 20.3 $\mathrm{fb}^{-1}$ of $pp$ data collected at $\sqrt{s} = 8~\mathrm {TeV}$, topological clustering used a fixed pile-up noise corresponding to $\langle \mu \rangle =30$. Fluctuations of pile-up due to different luminosity conditions as well as global and local event pile-up fluctuations can still affect the seeding and growth of clusters and require jet-level pile-up corrections.

The jet-area pile-up subtraction method reduces global fluctuations of pile-up in jets and allows the correction of jet shape variables. This method uses a direct measure of the pile-up activity in the calorimeter on an event-by-event basis (the $p_{\text {T}}$ density $\rho$ in $\eta$–$\phi$ space), as well as a jet-by-jet measure of pile-up susceptibility (the jet area, $A^{\mathrm {jet}}$). A residual pile-up correction is necessary to fully accommodate the impact of pile-up on jet $p_{\text {T}}$ as the high-occupancy jet core contributes some extra sensitivity to both in-time and out-of-time pile-up, and the effects of pile-up on forward jets are not fully described by the median $p_{\text {T}}$ density as calculated from topological clusters in the central calorimeter. The combination of $\rho \times A^{\mathrm {jet}}$ subtraction and residual correction results in a stable jet $p_{\text {T}}$ response across the full range of pile-up conditions in 2012, and it significantly reduces the degradation in jet $p_{\text {T}}$ resolution associated with fluctuations in pile-up. It also reduces the dependence of jet multiplicity on pile-up, shifting the majority of pile-up jets below the minimum jet $p_{\text {T}}$ threshold. For $p_{\text {T}} > 50~\mathrm {GeV}$, the pile-up subtraction procedure alone is sufficient to make the jet multiplicity stable as a function of $\langle \mu \rangle$ and $N_{\mathrm{PV}}$ within statistical errors. Systematic uncertainties are typically below $2~\%$ for $R = 0.4$ anti-$k_{t}$ jets with $p_{\text {T}} > 40~\mathrm {GeV}$ in the central region of the calorimeters; they reach up to $6~\%$ at low $p_{\text {T}}$ and higher $\eta$. Jet-area subtraction also significantly reduces the pile-up dependence of jet shape variables.

Jet vertex tagging enables the identification and rejection of pile-up jets arising from local fluctuations of pile-up within events, as well as from QCD jets originating from pile-up vertices. A fundamental feature of the $\mathrm{JVT}$ algorithm, introduced in this paper, is that its discrimination power is independent of the pile-up conditions, leading to hard-scatter jet selection efficiencies that are stable within $1\%$ for up to 35 interactions per bunch crossing. This pile-up stability implies that there is no need to re-optimise selections based on $\mathrm{JVT}$ as pile-up conditions change, even as the LHC transitions to $\sqrt{s} = 13~\mathrm {TeV}$ and 25 ns bunch spacing in Run 2. The JVT selection efficiency, measured as a function of $p_{\text {T}}$ and $\eta$, is found to agree between data and simulation within 1–2 %.

Jet vertex tagging has also been extended to the case of large-$R$ jets by introducing a track-based trimming algorithm at the subjet level. The new track-based grooming achieves performance similar to that of calorimeter-based trimming, while using complementary tracking information. In particular, track-based grooming does not need to rely on subjet $p_{\text {T}}$ selection cuts as in the case of standard grooming methods. Jet cleansing has also been studied and results in performance similar to that of all other methods considered.

The suite of algorithms discussed in this paper has provided the capability to manage and suppress pile-up, both at the level already observed during the LHC Run 1 and at the level expected for Run 2. The impact on jet reconstruction and measurement is significant and has thus improved many aspects of the physics program in ATLAS. Pile-up corrections and suppression algorithms both for small and large radius jets have enhanced the discovery potential of the ATLAS experiment and improved the precision for Standard Model measurements. New and more advanced methods that are presented in this paper and developed towards the end of the LHC Run 1 will provide additional handles and improved precision for pile-up mitigation for the upcoming LHC Run 2 and the future high-luminosity upgrades.

## Footnotes

1 The ATLAS reference system is a Cartesian right-handed coordinate system, with the nominal collision point at the origin. The anticlockwise beam direction defines the positive *z*-axis, while the positive *x*-axis is defined as pointing from the collision point to the centre of the LHC ring and the positive *y*-axis points upwards. The azimuthal angle $\phi$ is measured around the beam axis, and the polar angle $\theta$ is measured with respect to the *z*-axis. Pseudorapidity is defined as $\eta = - \ln [\tan (\theta /2)]$, rapidity is defined as $y = 0.5\ \ln [(E + p_z)/(E - p_z)]$, where *E* is the energy and $p_z$ is the *z*-component of the momentum, and the transverse energy is defined as $E_{\text {T}} = E \sin \theta$.

2 Tracks are assigned to vertices by requiring $| \Delta z \times \sin \theta | < 1$ mm. In cases where more than one vertex satisfies this criterion, ambiguity is resolved by choosing the vertex with the largest summed $p_{\text {T}} ^2$ of tracks.

3 With this particular choice of *k*, the resulting $\mathrm{corrJVF}$ shapes for hard-scatter and pile-up jets are similar to the corresponding ones for $\mathrm{JVF}$.
